# Black perithecial pigmentation in *Fusarium* species is due to the accumulation of 5-deoxybostrycoidin-based melanin

**DOI:** 10.1038/srep26206

**Published:** 2016-05-19

**Authors:** Rasmus J. N. Frandsen, Silas A. Rasmussen, Peter B. Knudsen, Silvio Uhlig, Dirk Petersen, Erik Lysøe, Charlotte H. Gotfredsen, Henriette Giese, Thomas O. Larsen

**Affiliations:** 1 Department for Systems Biology, Technical University of Denmark, DK-2800 Kgs. Lyngby, Denmark; 2 Section for Chemistry and Toxicology, Norwegian Veterinary Institute, Oslo, Norway; 3 Department of Chemistry, University of Oslo, Oslo, Norway; 4 Department of Plant Health and Biotechnology, NIBIO - Norwegian Institute of Bioeconomy Research, Høgskoleveien 7, 1430 Ås, Norway; 5 Department of Chemistry, Technical University of Denmark, DK-2800 Kgs. Lyngby, Denmark; 6 Department of Chemistry and BioScience, AAU, Fredrik Bajers Vej 7, 9220 Aalborg, Denmark

## Abstract

Biosynthesis of the black perithecial pigment in the filamentous fungus *Fusarium graminearum* is dependent on the polyketide synthase PGL1 (*oPKS3*). A seven-membered *PGL1* gene cluster was identified by over-expression of the cluster specific transcription factor *pglR*. Targeted gene replacement showed that *PGL1, pglJ, pglM* and *pglV* were essential for the production of the perithecial pigment. Over-expression of *PGL1* resulted in the production of 6-O-demethyl-5-deoxybostrycoidin (**1**), 5-deoxybostrycoidin (**2**), and three novel compounds 5-deoxybostrycoidin anthrone (**3**), 6-O-demethyl-5-deoxybostrycoidin anthrone (**4**) and purpurfusarin (**5**). The novel dimeric bostrycoidin purpurfusarin (**5**) was found to inhibit the growth of *Candida albicans* with an IC_50_ of 8.0 +/− 1.9 μM. The results show that *Fusarium* species with black perithecia have a previously undescribed form of 5-deoxybostrycoidin based melanin in their fruiting bodies.

The sexual development of the homothallic *Fusarium graminearum* (*Fg*) on wheat plants and in culture is well-described^1^,^2^. Perithecia (fruiting body) formation can be induced *in vitro* by cultivating the fungus on special media, typically based on plant material, such as carrot^3^. The mature perithecia are flask-shaped, 140–200 μm in diameter, with an ostiole at the top^2^. The periderm of the perithecia consists of three layers distinguishable by light microscopy. The outer layer is two to three cells thick and consist of thick-walled, highly vacuolated spherical cells that accumulate a blue-violet pigment of unknown structure^2^ (Fig. 1). This pigment gives the perithecia their black appearance on the macroscopic scale, a feature that is shared by all members of the former *Gibberella* genus^4^. The function of the pigment is unknown, but protection of the ascospores inside the perithecium from UV radiation and reactive oxygen species or inhibition of ascospore germination has been suggested^5^,^6^,^7^,^8^.

Disruption of the 15 type 1 iterative polyketide synthase (PKS) encoding genes in *Fg* PH-1, has previously shown that o*PKS3* (*PGL1*) was essential for production of the blue-violet perithecial pigment^7^. The *PGL1* gene is under a tight regulation in *Fg,* and expression is detected during late perithecium development coinciding with black pigmentation^7^,^9^,^10^,^11^. Deletion of the *PGL1* ortholog in *Fusarium verticillioides* (*Fve*, former *Gibberella* member) also resulted in albino peritheica^5^. Proctor *et al*. suggest that the *PGL1* gene is part of a gene cluster, based on gene synteny in the genomes of *Fg, Fve* and the more distantly related *Fusarium solani* (*Fs*). *Fs* and other members of the former *Nectria* genus are characterized by their red perithecia, a trait that has been linked to the activity of *pksN* and not *PGL1*^6^. The product of *Fs* PGL1 has been shown to be 3-acetonyl-1,6,8-trihydroxy-2-naphthaldehyde (6-O-demethylfusarubinaldehyde) by heterologous expression of the gene in *Aspergillus oryzae*^12^. This compound display a cyclization pattern identical to the pattern predicted for formation of fusarubins and bostrycoidins, both well-characterized mycelium pigments in *Fs*^12^. The identified primary product of PGL1 was also found in *Fusarium fujikuroi* (*Ff*, formerly *Gibberella*)^13^ where deletion of the *PGL1* ortholog (*Ff_fsr1*) caused loss of various fusarubin metabolites in the mycelium under perithecium inductive conditions. The deletion also led to loss of perithecial pigmentation, and the authors hence concluded that fusarubin is also responsible for perithecial pigmentation. Fusarubins are characterized by their yellow to red colors at physiological pH^14^, which does not correspond to the color observed in *Fg* perithecia and other former members of the *Gibberella* genus. This indicates that PGL1 might produce alternative compounds to fusarubin in perithecial tissues. Since the previous studies have not included chemical analysis of perithecia the aim of this study has been to reveal the chemical composition of the pigments in perithecia from *Fusarium* sp.

## Results

### The analyzed Fusaria sp. all contain the PGL cluster

**Figure 2:**
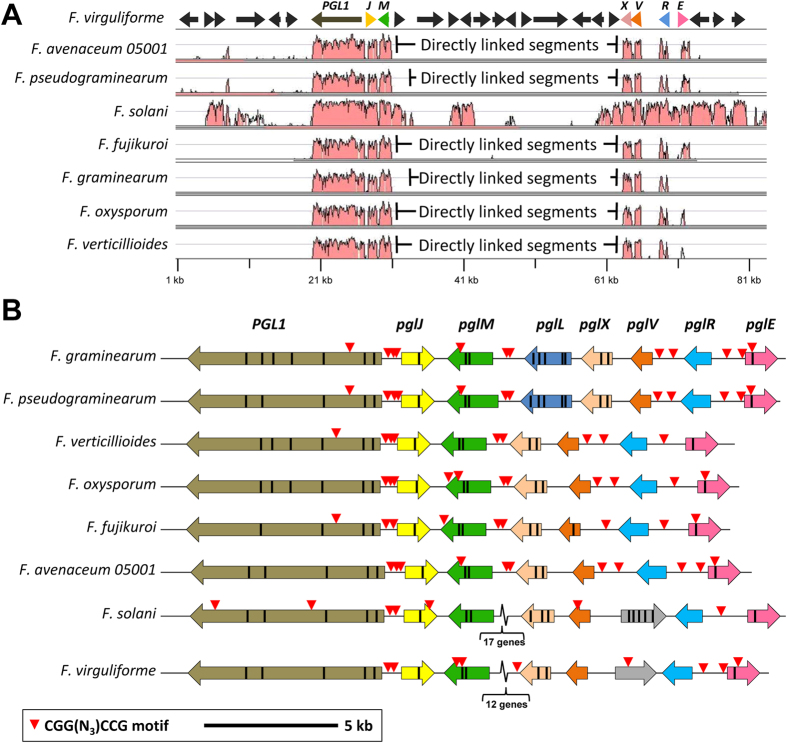
Identification of the core *PGL1* gene cluster and possible transcription factor binding sites. (**A**) Shuffle-LAGAN alignment of the putative *PGL1* gene cluster from eight different fusaria species with *Fvi* as a reference sequence (top). The graphs show the level of %-identity between the given species and *Fvi*, shown from 50% to 100% calculated using a 100 bp sliding window. (**B**) The core *PGL1* gene clusters consisting of *PGL1, pglJ, pglM, pglX, pglV, pglR* and *pglE. Fg* and *Fp* contains an additional gene *pglL*, larger inserts are found at the same site in the *Fs* and *Fvi* clusters (*Fs* contain a duplication of the *Fvi* sequence). Analysis of the promoter regions identified a single palindromic sequence (CGGN_3_CCG) to be significantly enriched. The location of this potential transcription factor binding motif is largely conserved across the different species, except for *Fs* and *Fvi* where none is found in the *plgM* and *plgV* promoters.

Comparative genomics of eight genome sequenced *Fusarium sp.,* using Shuffle-LAGAN alignment of the genomic regions surrounding the *PGL1* loci, revealed extensive sequence synteny (Fig. 2A). The syntenic region included seven genes, found in all analyzed species, suggesting that the core *PGL1* gene cluster consist of *PGL1, pglJ, pglM, pglX, pglV, pglR* and *pglE* (Fig. 2B). The relative orientation of the genes was conserved across the species. However, *Fg* and *Fusarium pseudograminearum* (*Fp*) contained a 2.3 kb insert located between the *pglM* and *pglX* genes (Fig. 2B), a region that includes *Fg-pglL. PglL* is predicted to encode adenylosuccinate lyase (Ade13), a key enzyme in the central metabolism. As no other orthologs were found in the *Fg* genome we hypothesize that *pglL* is not part of the cluster^15^. In *Fs* and *Fusarium virguliforme* (*Fvi*), both members of the former *Nectria* genus, larger inserts are also found between *pglM* and *pglX* (Fig. 2A). The insert in *Fs* is approximately twice the size of that found in *Fvi,* and a dot-plot based comparison revealed that the *Fs* sequence contained a partial duplication of the *Fvi* sequence. It is currently impossible, based on the available data, to determine whether the inserts found in the *Fg, Fp, Fs* and *Fvi* clusters represent evolutionary steps towards assembly or breakup of the cluster.

Analysis for functional domains in the encoded enzymes (Supplementary file Table S1) yielded very similar results to those reported by Brown and co-workers^16^.

### pglR encodes a PGL1 cluster-specific transcription factor

**Figure 3:**
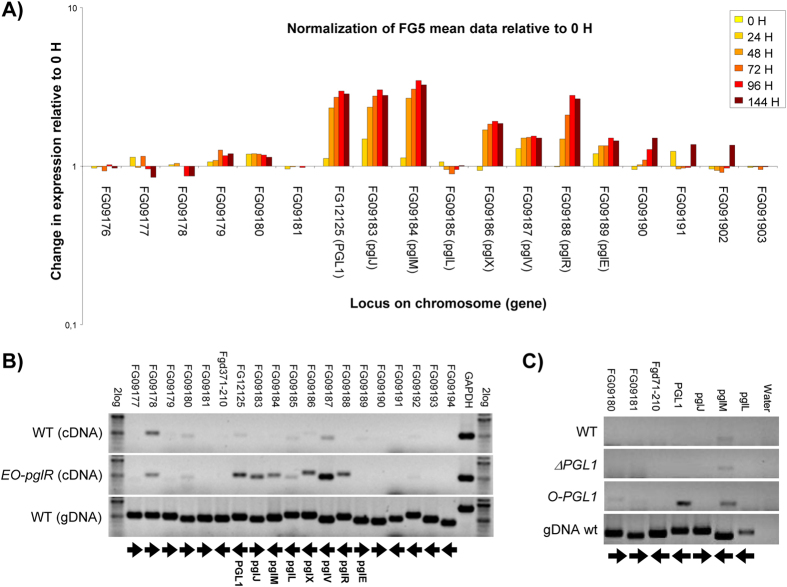
Expression analysis of the *PGL1* gene cluster and the effect of PglR overexpression on transcription in *Fg*. (**A**) Affymetrix gene expression: Change in gene expression as a function of the location on the genome with focus on the genes surrounding the *PGL1* gene. The change in expression level is shown relatively to 0 H for the six analyzed time points (0, 24, 48, 72, 96 and 144 h). (**B**) Expression analysis (RT-PCR) of the putative *PGL1* gene cluster in the wild type and *pglR* overexpression strain. The lower gel shows the positive control with genomic DNA from the wild type. The arrows under the gel show gene orientation in *Fg*. (**C**) Expression analysis (RT-PCR) of genes surrounding the *PGL1* locus in the *Fg* wild type, Δ*PGL1,* and O-PGL1 strains, with wild type gDNA as a positive control.

**Figure 4:**
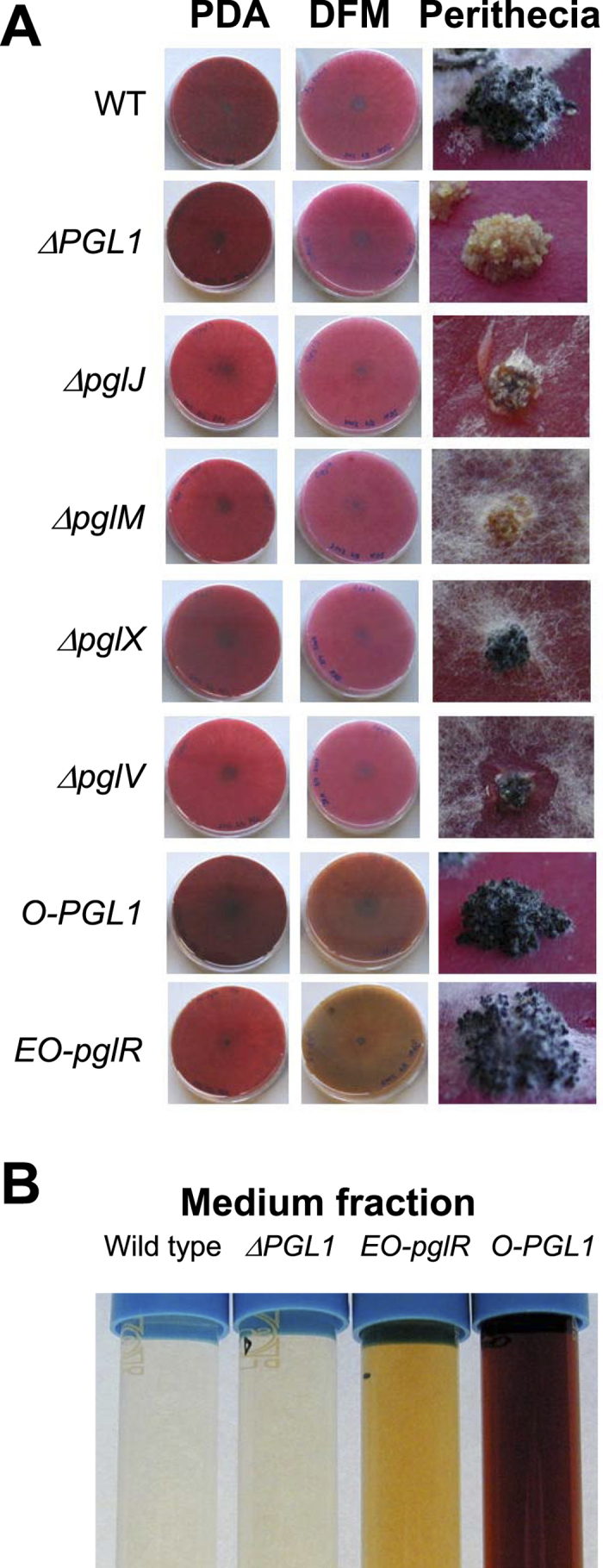
Phenotypes of the generated *Fg* strains. (**A**) Cultivated on solid DFM medium and perithecium formation on carrot agar. (**B**) Medium fraction from liquid cultures of wild type, Δ*PGL1,* EO*-pglR* and O*-PGL1* strains cultivated for ten days in liquid DFM. The medium was filtered and centrifuged to remove cell debris and non-soluble metabolites.

Analysis of the available Affymetrix expression data^9^ for perithecia development in *Fg* showed that six conserved genes: *pglM, pglJ, pglX, pglV, pglR,* and *pglE* were co-regulated with *PGL1* and that expression of this putative cluster peaked at 96 hours after induction of perithecium formation (Fig. 3A). The cluster includes a putative transcription factor encoding gene *pglR* (FG09188) (Table S1). The role of PglR in regulation of the cluster was examined by introducing an additional copy of the *pglR* under the control of the constitutive *Aspergillus nidulans GAPDH* promoter, into the *PKS12* locus. *Agrobacterium*-mediated transformation (AMT) of the expression cassette resulted in 45 EO-*pglR* transformants, of which two were verified by diagnostic PCR and Southern analysis (Supplementary File 1). Overexpression of *pglR* did not affect perithecia formation or color, but the vegetative mycelium displayed a yellow-brown pigmentation not observed in the white reference strain (FgHUEA:Δ*PKS12*) (Fig. 4A,B). Cultivation of the strain in liquid DFM media showed that the novel pigments were excreted and soluble in the aqueous solution.

Expression analysis of the putative *PGL1* gene cluster, by semi-quantitative RT-PCR, showed that *PGL1, pglM, pglJ, pglX, pglV* and *pglR* were up-regulated in vegetative mycelium of the EO-*pglR* strain compared to the wild type, while expression of *pglE* was not affected (Fig. 3B). These results show that PglR is the positive acting pathway specific transcription factor for the *PGL1* cluster consisting of the six genes.

### Identification of a putative binding motif for PglR

A search for potential palindromic transcription factor binding sites in the five promoter regions of PglR regulated genes resulted in the identification of a CGGN_3_CCG motif, which was significantly overrepresented (P = 2.5e-07), e.g. the motif occurred nine times (1.9 time/kb) in the promoters in the *Fg* cluster, while the background occurrence of the motif was 0.10 times/kb in all *Fg* promoters (Fig. 2B). Similar results were obtained for *Fusarium oxysporum (Fo*) and *Fve*. The motif was found in four of the five promoter regions, and the locations of these potential binding motifs were largely conserved across the different species (Fig. 2B), except in *Fs* and *Fvi* where none was found in the promoters of *plgM* and *plgV*. The conservation in placement of the motif and the significant overrepresentation suggests that the sites are under active selection, supporting a biological function.

### The O-PGL1 and EO-pglR strains produced novel compounds

Efforts to identify and characterize pigments directly from perithecia failed as minute amounts were obtained. As an alternative we used genetic engineering to overexpress the involved genes in the vegetative mycelium of the fungus. To determine the primary product of *Fg-*PGL1 we exchanged the endogenic perithecial specific *Fg-PGL1* promoter with the constitutive *A. nidulans GAPDH* promoter. A total of 42 transformants were isolated of which two were verified by diagnostic PCR and Southern analysis (Supplementary File 1). Introduction of the constitutive promoter in front of *Fg-PGL1* resulted in expression of the gene in the vegetative mycelium, and had no detectable effects on the expression of the neighboring genes belonging to the cluster (Fig. 3C). The mycelium of the *O-PGL1* strain displayed a brown phenotype, which differed significantly from the red color of the wild type (Fig. 4A). The strain produced perithecia at a similar rate and appearance as the wild type (Fig. 4A). Cultivation of the *O-PGL1* strains in liquid media (DFM and YPG) resulted in a brown coloring of the culture broth, which was not observed for the wild type (Fig. 4B).

**Figure 5:**
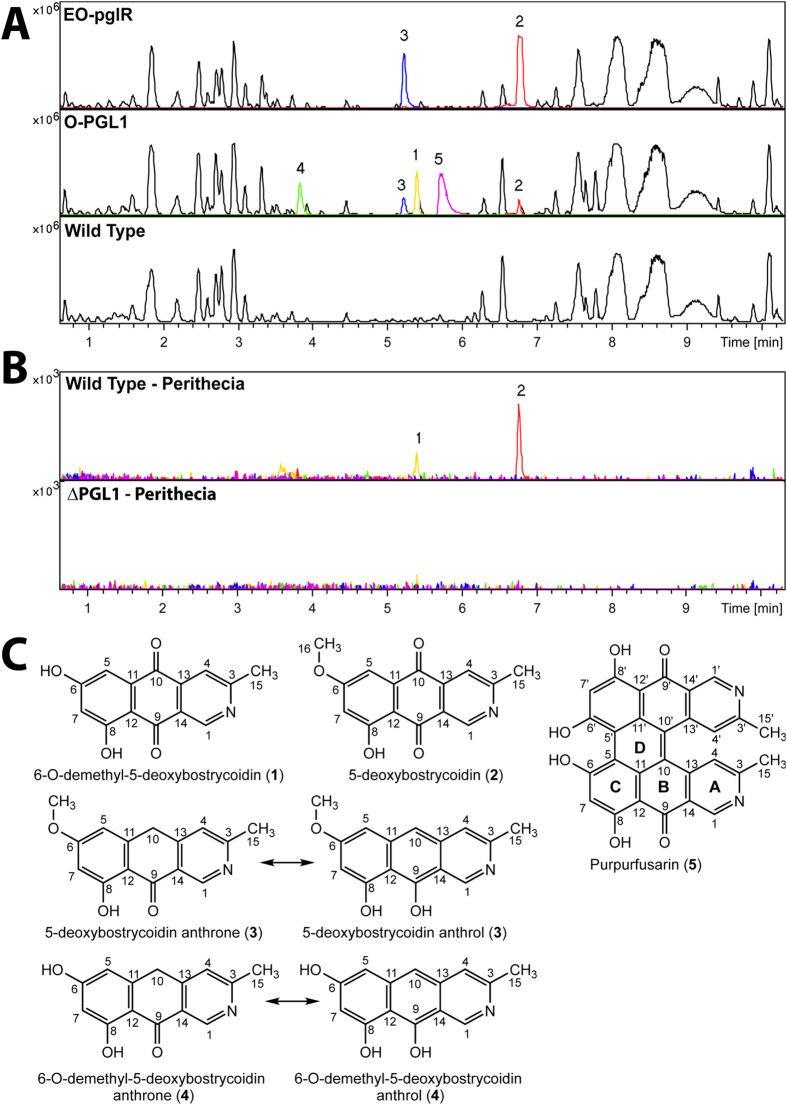
Chemical analysis and structures of purified compounds. (**A**) BPC comparing wild type to the EO-PGL1 and O-PGL1 strains. Overlaid with the EIC of **1** (Yellow), **2** (Red), **3** (Blue), **4** (Green) and **5** (Purple). (**B**) Bottom panel shows EIC for compound 1–5 from the analysis of perithecia from the wild type and Δ*PGL1* strain. Compound 1 and 2 were detected in perithecia from the wild type but not the Δ*PGL1* strain. (**C**) Structures of the identified compounds 1–5.

UHPLC-DAD-HRMS based analysis of the filtered culture broth, from 7-day old O-*PGL1* and EO-*pglR* cultures, revealed five compounds not present in the wild-type (Fig. 5A). Compounds (**2**) and (**3**) were found in the EO-*pglR* strains, while the O-PGL1 strain produced all five compounds (**1**)**–**(**5**). The elemental composition and UV spectra of the five compounds all suggested highly conjugated compounds, consistent with that of polyketide derived pigments. Extracted ion chromatograms for the five identified compounds showed that the wild-type did not produce any of the compounds in the vegetative mycelium. This supports that production of the five novel compounds in the overexpression strains were the result of switching on one or more of the *PGL1* genes. Dereplication resulted in tentatative identification of compound (**1**) and (**2**) as 6-O-demethyl-5-deoxybostrycoidin (**1**) and 5-deoxybostrycoidin (**2**), respectively. Their structures were later confirmed by NMR spectroscopy, comparing the obtained data with those found in the literature^17^. In addition to compound (**1**)**–**(**5**) the concentration of several other compounds was found to increase in the mutants compared to the wild type strain (Fig. 5A). Extracted ion chromatograms of these compounds showed that they were all produced at low concentrations in the vegetative mycelium of the wild type. This indicates that their production is independent of the *PGL1* gene which is silent in the vegetative mycelium of the wild type (Fig. 3C).

### Structural elucidation of 5-deoxybostrycoidin anthrone (3)

The effort to identify compound (**3**) by dereplication was unsuccessful. Its [M+H]^+^ was found to be 256.0965 m/z, and its elemental composition was calculated to C_15_H_13_NO_3_ (theoretical [M+H]^+^ 256.0968). Examination of the 1D NMR showed that all signals had a minor peak in a ratio of 3:1.8. The reported structure is that of the major peaks. The 1D ^1^H-NMR consisted of one aromatic methyl group at 2.64 ppm (H-15), one methyl ether group at 3.87 ppm (H-16), four aromatic protons at 6.24 (H-7), 6.44 (H-5), 7.17 (H-4) and 9.35 (H-1), one aromatic hydroxyl at 13.17 ppm (OH-8) and lastly two protons at 4.24 ppm (Table S4). In addition, the two protons H-5 and H-7 appeared to be meta-coupled (J = 2 Hz). The main difference in the 1D NMR spectra between the proposed structure of (**3**) and the structure of (**2**) was found in the signal of H-10, this signal at 4.24 ppm and C-10 at 31.5 ppm was found to be in good agreement with what has been reported for emodin anthrone^18^. The minor peaks were consistent with the tautomer of (**3**) the 5-deoxybostrycoidin anthrol (Fig. 5C).

### Structural elucidation of 6-O-demethyl-5-deoxybostrycoidin anthrone (4)

From the O-PGL1 strain an unknown compound with a [M+H]^+^ of 242.0711 m/z was isolated. The chemical formula was calculated to C_14_H_11_NO_3_ (theoretical [M+H]^+^ 242.0812). The UV spectrum was identical to that of (**3**). In addition, the chemical formula only differed by a single CH_3_ suggesting (**4**) to be the 6-O-demethylated form of (**3**) (Fig. 5C). Due to very low amounts of the compound, only 1D NMR was performed. The examination of the 1D NMR of (**4**) in DMSO-*d6* showed one aromatic methyl group at 2.32 ppm (H-15), five aromatic signals at 5.93 (H-5 or H-7), 6.25 (H-7 or H-5), 6.31 (H-10 or H-5), 6.90 (H-5 or H-10), 8.64 (H-1) and two aromatic hydroxyl groups at 9.91 and 15.43 ppm. The small coupling constant (*J* = 1.8 Hz) for the two doublets at 5.92 and 6.25 ppm suggest that these are meta substituted. No O-methyl group signal was observed in the range of 3–4 ppm confirming the demethylation at position OH-6. Nor was a methylene signal observed around 4 ppm suggesting that (**4**) has adopted the anthol conformation in DMSO as deduced in Fig. 5C.

### Structural Elucidation of Purpurfusarin (5)

The purple compound (**5**) was only observed in the O-PGL1 strain. The monoisotopic m/z of its [M+H]^+^ ion was 477.1080 Da, corresponding to an elemental composition of C_28_H_16_N_2_O_6_ (theoretical [M+H]^+^ 477.1081 m/z). Examination of the ^1^H spectrum revealed 6 singlet resonances: one aromatic methyl group at 2.37 ppm (H-15/15′), three aromatic resonances at 6.39 (H-7/7′), 7.72 (H-4/4′) and 9.43 (H-1/1′) ppm and two phenolic resonances at 15.86 (OH-8/8′) and 18.47 (OH-6/6′) ppm (Table S5). An interesting observation was that the extreme downfield hydroxyl proton had an odd signal intensity ratio to the aromatic protons and methyl group being 1:2:6. This suggested that this hydroxyl group was partly deprotonated or exchanged. Examination of the ^13^C spectrum revealed 14 resonances, all of which could be accounted for in the HMBC and HSQC. The mass spectrometric analysis gave an elemental composition of the molecule with 28 carbon atoms, (**5**) indicating a highly symmetrical compound. The aromatic proton signals at 7.72 and 9.43 indicated the proximity of an aromatic nitrogen, which was confirmed by 15N-HMBC: both H-1/H-1′ and H- 4/H-4′ as well as the methyl protons at 2.73 ppm correlated to a nitrogen at 317.5 ppm (relative to external liquid ammonia at 25 °C).

The elucidation of (**5**) was aided by the observed similarity between ^1^H and ^13^C shifts of (**1**) and (**5**) (Table S5) and those reported for bostrycoidin^19^. Especially, the chemical shift values in the nitrogen containing ring were directly comparable. As the NMR and HRMS suggested a dimer, we hypothesize that (**5**) is a fusion product of the molecules (**1**) and (**4**). The fusion of two anthrones via oxidative coupling has similarly been described as a putative mechanism in the biosynthesis of protohypericin and hypericin found in St. John’s wort^20^. The oxidative coupling of two anthrones could give rise to two different isomeric configurations, C1 and C2 as shown in Supplementary File 1. However, the SELNOE (1D selective NOESY) spectra made by individual irradiation of all ^1^H-resonanances except for the two downfield hydroxyl protons, and the 2D NOESY spectra, showed (merely) a strong correlation between the methyl group H-15/H-15′ and H-4/H-4′ and a comparatively weak correlation between H-15/H-15′ and H-1/H-1′, while no correlation between H-4/H-4′ and H-7/H-7′ or H-4/H-4′and 6/6′-OH could be observed, which would have been expected if 5 had adopted the isomeric configuration C2 Hence, we expect (5) to adopt the C1 configuration. This is further supported by Falk *et al*.^20^ reporting that titrating hypericin with KOH generated a hypericinate ion with a characteristic hydroxyl resonance at 18.37 ppm exhibiting a 1:2:6 signal intensity ratio, similar to what we observed in (**5**). Hence, we suggest that (**5**) adopt the C1 configuration as seen in protohypericin (Fig. 5C).

### PGL1, pglJ, pglM and pglV are involved in perithecial pigment production

Targeted replacement of *pglJ, pglM, pglV, pglX* and *PGL1* by AMT resulted in 40 Δ*PGL1*, 42 *O-PGL1*, 15 Δ*pglJ*, 21 Δ*pglM*, 30 Δ*pglV* and 39 Δ*pglX* hygromycin resistant transformants. The PCR and Southern based analysis of the isolated transformants verified the desired modification and single copy integration of the T-DNA (Supplementary File 1). For each mutant type, a single verified transformant was selected for further phenotypical characterization. The Δ*PGL1* strain produced white perithecia on carrot agar plates (Fig. 4A), as previously reported by Gaffoor *et al*.^7^. Targeted replacement of *pglJ, pglM* and *pglV* resulted in perithecia, with an altered pigmentation compared to the wild type (Fig. 4A). The Δ*pglJ* and Δ*pglM* strains had light yellow perithecia, and the Δ*pglV* strain dark brown perithecia. Replacement of *pglX* did not visually affect perithecium pigmentation. Chemical analysis, by targeted UHPLC-DAD-HRMS of isolated perithecia from the generated Δ*PGL1* deletion strain, supported the visual observations (Fig. 5B). Compounds (**1**) and (**2**) were detectable (EIC view) in extracts from the wild type perithecia while neither was found in the Δ*PGL1* strains. A similar search for fusarubin based metabolites did not reveal any peaks, showing that the blue-violet pigments in the *Gibberella* type of perithecia must be based on bostrycoidins and not fusarubin metabolites.

### Antifungal susceptibility testing

The IC_50_ of (**5**) and the positive reference yanuthone D for *C. albicans* was extrapolated from compound specific dilution sequences and annotated as the average concentration for which 50% inhibition plus minus the standard deviation was observed. Purpurfusarin was found to have an IC_50_ of 8.0 +/− 1.9 μM and yanuthone D with a IC_50_ of 3.3 +/− 0.5 μM.

## Discussion

The *PGL1* gene cluster was found to consist of six core genes that were highly conserved in the analyzed *Fusarium* species (Fig. 2B). Overexpression of *PglR* confirmed that the cluster was regulated by PglR, as also described for *Ff*^13^. Analysis of promoter regions in PglR co-regulated genes revealed a significant enrichment of the CGG-N_3_-CCG motif. The Zn(II)_2_Cys_6_ family of transcription factors often bind to short palindromic sequences, consisting of inverted trinucleotide repeats separated by a variable length spacer^21^. The identified CGG repeat is also seen in a number of other Zn(II)_2_Cys_6_ transcription factors, such as GAL4 (CGG-N_11_-CGG)^22^, War1 (CGG-N_23_-CCG)^23^ and Rds1 (CGGCCG)^24^, which suggest a common mode of protein-DNA interaction and that PglR binds as a homodimer. The overrepresentation and the conserved position across the species make the motif a strong candidate for a PglR binding motif.

**Figure 6:**
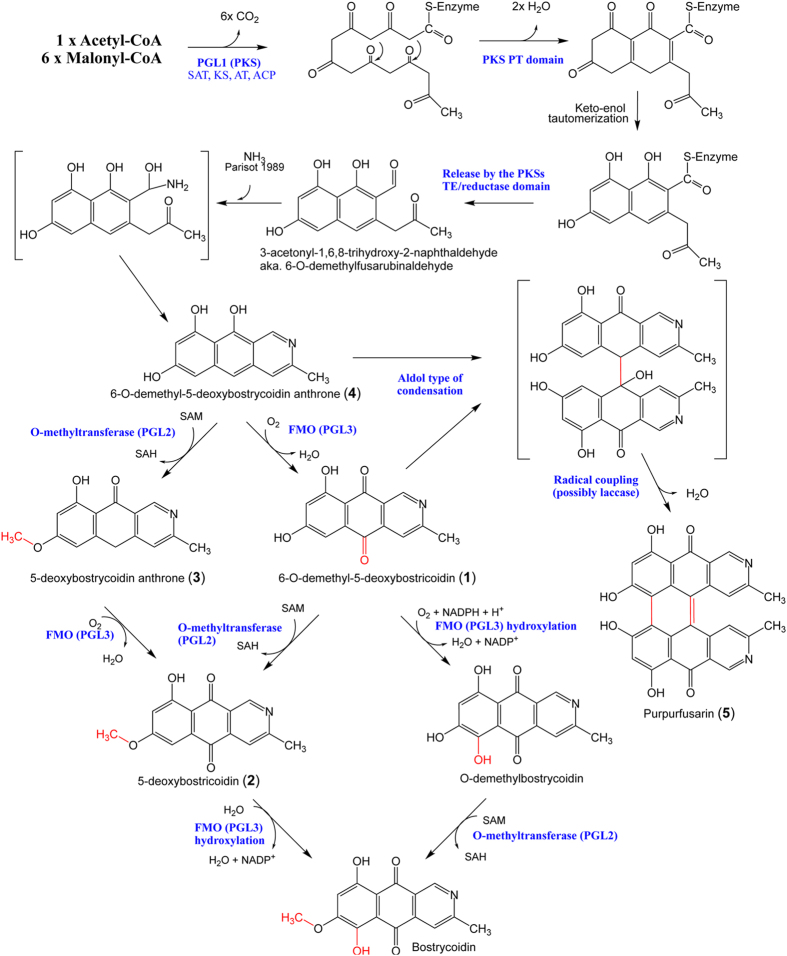
Proposed biosynthetic pathway for the formation of 5-deoxybostrycoidin. Compounds in brackets are predicted intermediates. Compound 3 and 5 are likely shunt products formed upon expression in the mycelium tissue.

Targeted deletion of *PGL1, pglJ, pglM* or *pglV* in *Fg* impacted the perithecium color (Fig. 4A), showing that the encoded enzymes are required for biosynthesis of the pigment. Transcriptional activation of *PGL1* resulted in the formation of five pigments (**1**)**–**(**5**) in the mycelium, while activation of the entire cluster by *pglR* overexpression led to the accumulation of (**2**) and (**3**). The identified compounds all belong to the bostrycoidin family that has not previously been reported in *Fg,* and three of the formed compounds (**3–5**) are completely novel. Based on the structure of the identified compounds and the biosynthetic potential of the involved enzymes, we formulated a model for the biosynthetic pathway (Fig. 6). This includes two alternative routes for the formation of (**2**), the most decorated of the compounds. Based on the results of Awakawa *et al*. the primary product of PGL1 was expected to be 6-O-demethyl-fusarubinaldehyde^12^. However, the simplest compound detected in the O-*PGL1* strain was the nitrogen-containing compound (**4**). Standard polyketide biosynthesis does not offer an explanation for the introduction of nitrogen as observed in bostrycoidins. Parisot *et al*. have, however, previously shown that bostrycoidins can be formed at room temperature from ‘anhydrofusarubin lactol’ when reacting with ammonia (50% after 72 hours at room temperature), making it likely that the compound is also formed spontaneously *in vivo*^25^. Alternatively, the nitrogen atom is introduced by aminotransferase activity that transfers the amine group into the terminal aldehyde of 6-O-demethyl-fusarubinaldehyde similarly to what has been proposed by Wagoner *et al*.^26^. Formation of (**1**), (**2**), and (**3**) in the *O-PGL1* strains shows that the vegetative mycelium contains enzymes that are capable of converting the primary PKS product (**4**) to (**1**)**–**(**3**). Expression of the entire *PGL1* gene cluster eliminated accumulation of the early pathway intermediates (**1**) and (**4**), showing that the cluster encoded enzymes can outcompete the shunt reaction in the mycelium. We propose that the linking of compounds (**1**) and (**4**) to yield the dimeric compound (**5**) is likely to proceed via an aldol type of condensation, followed by generation of the core double bond by loss of water and finally phenolic oxidative coupling (Fig. 6), possibly catalyzed by the GIP1 laccase from the aurofusarin gene cluster, which is known to be active in the mycelium and modifies compounds with similar structural features^27^.

Though (**5**) was the only compound with a blue-violet color, similar to that found in perithecia, it was not itself detectable in perithecia (Fig. 5), suggesting that it is a shunt product only formed as a result of the modified expression pattern. However, the detection of (**1**) and (**2**) in wild-type perithecia suggests that these form the basis for formation of the unextractable blue-violet pigment, which could be either a polymer or compounds covalently linked to the cell wall. A situation that resembles what has been reported for other fungal pigments, such as DHN-melanin^28^.

Many of the intermediates from the fusarubin/bostrycoidin pathways have previously been shown to display a wide range of biological activities, which include antibiotic, fungicidal, insecticidal and herbicidal activities, reviewed by Parisot *et al*.^14^. As part of a larger screen, we tested the bioactivity of (**5**) against *C. albicans* and found that it had an IC_50_ of 8.0 +/− 1.9 μM; in comparison the positive standard (yanuthone B) in the experiment had an IC_50_ of 3.3 +/− 0.5 μM, while the less potent of the tested compounds had an IC_50_ >100 μM^29^. The available amounts of the two other novel compounds, (**3**) and (**4**), did not allow for a similar test.

*Fusarium sp.* are characterized by producing either of three different red mycelium pigments; aurofusarin (*oPKS12/AUR*), bikaverin (*oPKS16/BIK1*) or fusarubin (*oPKS3*/*PGL1*). The identified role of the *PGL1* gene cluster in *Fg* perithecia pigmentation and its formation of 5-deoxybostrycoidin likely extends to other members of the former *Gibberella* genus. The role of the cluster in mycelium fusarubin formation, in members of the former *Nectria* genus^6^, indicates that the cluster has undergone a dramatic shift in function during evolution. *PGL1* orthologs are found in all genome sequenced *Fusaria sp.,* while the PKS (*pksN*) responsible for the red perithecium pigment is only found in members of the former *Nectria* genus (*Fvi*: contig AEYB01000515 and AEYB01000516).

This high level of diversity, with respect to the combinations of pigments and their production patterns, is surprising. It could be argued that difference in pigment use is due to adaptation to different ecological niches, but species with different pigment profiles have been shown to inhabit the same niches^30^. A more plausible hypothesis is that the various polyketide pigments (fusarubin, bostrycoidin, bikaverin, aurofusarin and the uncharacterized red pigment from *Nectria* perithecia) are functionally redundant as they are all naphthoquinones capable of redox cycling and have overlapping absorption spectra. Functional redundancy based on two or more genes efficiently eliminates active selection on the genes. Nowak and coworkers have identified several evolutionary scenarios that allow for persistence of redundant genes, but in the majority of cases this situation results in random elimination of either of the redundant genes (systems) or a split of the shared function between the involved genes, to yield a genetically stable situation. The split can be accommodated by adapting different expression patterns, with respect to time or tissue, or by the evolution of novel non-overlapping functions^31^. The occurrence of the *PGL1* cluster in all sequenced *Fusaria* sp. suggests that this is the ancestral pigment system, compared to the *oPKS12, oPKS16,* and *pksN* systems that are only found in subclades of the *Fusarium* genus. The *PGL1* gene cluster may originally have been responsible for both mycelium and perithecium pigmentation, but the acquisition of a redundant pigment system led to a change in the *PGL1* clusters expression to accommodate two parallel pigment biosynthetic pathways for mycelial and perithecium pigmentation. Subsequent introductions of new pigment systems would result in replacement of old functionalities, see evolutionary models in Supplementary File 1. The alternative pigment systems were likely acquired by horizontal gene transfer events, involving entire gene clusters. The ‘division of labor’ model is supported by the observation that deletion of *oPKS12, oPKS16, PGL1* and *pksN* all result in albino tissues^5^,^6^,^13^,^32^,^33^, and that reported expression of the *Ff-PGL1* cluster in the *Ff* mycelium occurs under conditions where the normal mycelium pigment bikaverin is not produced^13^. The ‘division of labor’ model does not depend on absolute division to be genetically stable, but simply that the individual components each have one unique non-overlapping function, which would allow for situations where multiple pigment systems are active in the same tissues.

The conservation, replacement and development of redundant pigment systems strongly indicates that pigmentation plays a key to the survival of members of the *Fusarium* genus.

## Conclusion

The present study for the first time provides direct evidence that the black perithecial pigmentation in *Fusarium graminearum* is due to the accumulation of a 5-deoxybostrycoidin based melanin, and not as previously proposed fusarubins. A situation that likely extends to other *Fusarium* sp. with black perithecia, e.g. members of the former *Gibberella* genus. Synthesis of the detected 5-deoxybostrycoidin is based on a six-membered gene cluster, expression is controlled by the pathway specific transcription factor PglR. The study also offers an insight into the evolutionary forces that has shaped secondary metabolism of filamentous fungi in general. The existence of highly diverse pigment systems within the *Fusarium* genus can likely be explained by multiple horizontal gene transfers, involving entire biosynthetic gene clusters, resulting in genetic instability due to functional redundancy between the clusters. A situation that has either been resolved by random elimination of one of the clusters, or by changes in the clusters expression patterns to yield a genetically stable state. This model offers an explanation for how secondary metabolite gene clusters, in general, can be acquired and lost in an evolutionary perspective.

## Experimental Procedures

### Microorganisms, Culture conditions and Genetic modifications

*F. graminearum* PH-1 (NRRL 31084) wild-type was used as starting material for genetic modifications. *Agrobacterium tumefaciens* LBA4404 was used for *Agrobacterium* Mediated Transformation (AMT)^34^.

Vectors for targeted replacement (pAg1-H3::ΔPGL1) and overexpression (pAg1-H3E::O-PGL1) of *PGL1* were constructed by In-Fusion cloning^35^, using the primers described in Table S2. Vectors for targeted replacement of *pglJ, pglM, pglX* and *pglV* were constructed via USER cloning as described in Frandsen *et al*.^36^. The inserts were verified by sequencing. The vector for overexpression of *Fg-pglR* was constructed by PCR amplifying the genes coding sequence and terminator using the primers pglR-E1/E2 (Figure S2), followed by USER cloning into pRF-HUEA^37^. AMT of *Fg* was carried out as described in Malz *et al*.^32^ with the modification described in Frandsen *et al*.^27^. Correct *Fg* transformants were identified by PCR-based screening, using four primer pairs (Table S2) and Southern analysis. Genomic DNA, for Southern analysis, was obtained following the procedure described in Malz *et al*.^32^.

Perithecia were produced on carrot agar plates. The cultures were incubated for seven days, at 20 °C with a continuous exposure to a mix of cool white light and fluorescent black light (Blacklight-blue F18W/BLB-T8 from SYLVANIA). Self-fertilization was induced by spreading 2 ml 2.5% aqueous Tween 60 per plate with a sterile Drigalsky spatula. The incubation continued for additional 10–14 days, to allow for perithecia formation.

### Enzymes, oligonucleotides, kits and apparatus

The PfuTurbo Cx Hotstart DNApol (Stratagene) was used for USER cloning and Taq DNApol (Sigma) for screening and RT-PCR reactions. Restriction enzymes and USER enzymes were from New England Biolabs. Primers were from Invitrogen and MWG. Vector DNA was prepared from liquid cultures using the Qiagen Miniprep Kit. PCR products were purified using the GE Healthcare GFX clean-up system. Sequencing was performed by GATC Biotech AG (Constance, Germany).

### Genome sequences and comparative analysis of the PGL1 cluster

Genome sequences for *Fg, Fve* and *Fo* were retrieved from FGDB at MIPS and http://www.broadinstitute.org/*Ff* from GenBank (HE613440) with annotations from MIPS^38^, *Fs* from Nh 2.0^39^, *Fp* CS3096 (AFNW00000000.1)^40^, *Fvi* (AEYB01000000)^41^ and *Fa* 05001 (Fa05001) from Genbank^42^. The sequences were handled using CLC Main Workbench 7.0 (Qiagen).

*PGL1* orthologs in the eight *Fusarium* genomes were identified by blastn analysis. The DNA sequences (60–100 kb) surrounding the *PGL1* locus were retrieved and aligned using the Shuffle-LAGAN global chaining algorithm^43^, with a sliding window of 100 bp and the conservation level set to >70%^44^. The results were visualized with the mVISTA browser (http://genome.lbl.gov/vista/). Prediction of conserved functional motifs were performed using the Conserved Domain Database (CDD), Pfam and BRENDA^45^,^46^.

### Identification of putative binding sites for pglR (PGLR) in the PGL1 gene cluster

The promoter regions of the genes in the putative cluster were analyzed for potential transcription factor binding sites using “Dyad-analysis” from “Regulatory Sequence Analysis Tool” (RSAT) (http://www.rsat.eu/)^47^. The background occurrence of motifs in promoters (1000 bp upstream sequence) of all the predicted genes in *Fg* (13,332 genes), *Fve* (14,179 genes) and *Fo* (17,735 genes) were analyzed using the “DNA pattern” program at RSAT.

### Expression analysis of genes surrounding the PGL1 locus

Affymetrix GeneChips for gene expression in *Fg* during perithecium development in culture (accession no. FG5) was retrieved from PlexDB^9^,^10^. The dataset included data for six time-points during perithecium development. Data for the 20 genes surrounding the *PGL1* locus were retrieved as RMA-normalized data, and divided by the value at 0 H to show the change in expression relative to vegetative growth.

Vegetative mycelium for gene expression analysis was produced by cultivating triplicates of the wild type, O-*PGL1* and EO-*pglR* strains for 5 days, in 50 ml liquid Yeast Peptone Dextrose (YPD medium) at 25 °C in 300 ml Erlenmeyer flasks at 150 rpm. The cultures were filtered through a Miracloth, and the mycelium was washed twice with sterile water, then frozen in liquid nitrogen. cDNA was synthesized as described in Malz *et al*.^32^. Primers, amplifying between 359 bp and 545 bp, were designed for locus FG09177.3 to FG09194.3 (Table S3). Genomic DNA from the wild type was used as a positive PCR control. PCR conditions were: 95 °C for 5 min, 25 × (95 °C for 30 sec, 60 °C for 30 sec, 72 °C for 1 min) and a 72 °C for 10 min. Expression of *Fg-GAPDH* was used as a reference gene to monitor the general gene expression level and to normalize the cDNA levels.

### UPLC-HRMS analysis of extracts

Analytical LC-MS was performed using a Dionex Ultimate 3000 ultra-high performance liquid chromatography (UHPLC, ThermoFisher, Waltham, MA) equipped with a diode-array detector (DAD) system hyphenated to a maXis G3 Oa-TOF mass spectrometer (Bruker Daltonics, Billerica, MA). Samples were introduced with an injection volume of 1 μl for mass spectrometric analysis and 5 μl for recording of UV/VIS data. The separation was performed on a reverse-phase Kinetex C18 column (100 × 2.1 mm, 2.6 μm, Phenomenex, Torrance, CA, US). The column temperature was maintained at 40 °C. The mobile phase consisted of MilliQ treated H_2_O (A) and ACN (B) both containing 20 mM formic acid (FA). The analytes were eluted using a linear gradient, at a constant flow of 400 μl min^−1^, having a starting composition of 10% B and increased to 100% B over 10 min. This composition was held for 3 min before returned to 10% B over 0.1 min, and held at this for 2.4 min to re-equilibrate the column.

The detection of the analytes was performed using an online DAD (Dionex Ultimate 3000), configured to detect from 200 to 600 nm, combined with an online maXis 3G Qq-Oa-TOF (Bruker Daltronics GmbH). The analytes were ionized using positive electrospray. The nebulizer gas was set to 2.4 bars; the drying gas flow was 12 ml/min and the drying temperature was 220 °C. The capillary voltage was 4.5 kV. The MS was set to scan in full scan mode with a mass range of 100–1000 m/z. The MS was calibrated using sodium formate (Fluka analytical grade) applying the Bruker HPC (High Precision Calibration) algorithm infused prior to each sample run. Dereplication was performed using AntiBase 2010 (Hartmut Laatsch, Wiley-VCH) and an in-house database containing 972 natural compounds.

### Purification of novel pigments produced by overexpression strains

Extraction and purification of Purpurfusarin (**5**): The O-PGL1 strain was grown in liquid YPD media for ten days. The cultivation broth was partitioned between EtOAc four times, and the combined organic layer was concentrated *in vacuo*. The crude extract (620 mg) was fractionated on a normal phase 10 g diol column (ISOLUTE, BIOTAGE, Uppsala, Sweden). The column was eluted using different solvent systems, 15 ml at a time, from heptane to MeOH (Tables S6–8): A purple band trailed and co-eluted together with other dark compounds. This fraction (67.2 mg) was then re-run on a diol column (10 g), this time using a binary EtOAc/MeOH gradient. The purple compound eluted in two fractions that were pooled (8.4 mg). The final purification of the purple compound was achieved on a Sedaphex LH-20 column (40 × 4 cm), equilibrated in MeOH. The column was eluted with MeOH with a linear flow rate of 3.2 cm/h. The separation was visually guided by the purple band. The pooled LH-20 fractions yielded 3.0 mg.

Extraction and purification of 5-deoxybostrycoidin anthrone (**3**): The EO-*pglR* strain was cultivated in liquid YPG media for ten days. The filtered broth was partitioned between EtOAc four times, and the combined phases were concentrated *in vacuo* given a dark yellow powder. The final purification of (**3**) was achieved on a LUNA PFP column (250 × 10 mm, 5 μm, Phenomenex, Torrance, CA, US). The column was eluted using a linear gradient consisting of MeOH and MilliQ H_2_O, both containing 20 mM FA. The gradient was 60–100% MeOH over 20 min. Two fractions were collected containing a yellow and a red compound. After evaporation of the solvent under a stream of nitrogen, the yellow fraction turned red. LC-HRMS of the two fractions showed that the compound eluted at the same times and the fractions were thus pooled, giving a total yield of 3.8 mg.

Extraction and purification of 6-O-Demethyl-5-deoxybostrycoidin anthrone (**4**): The O-PGL1 strain was cultivated in YPG liquid media for three days. The filtered broth was partitioned between EtOAc four times and the combined phases were concentrated *in vacuo*. The crude extract (144 mg) was fractionated on a 10 g Isolute Diol (see Table S for details). Compound (**4**) eluted in two fractions that were pooled to yield 33.8 mg. The final purification of (**4**) was achieved on a LUNA PFP column (250 × 10 mm, 5 μm, Phenomenex, Torrance, CA, US) in a 60–100% MeOH gradient containing 20 mM FA over 20 min. The yield was below 1 mg.

### Nuclear magnetic resonance (NMR) measurements

All 1D and 2D NMR experiments were acquired on a Varian Unity Inova 500 MHz (Varian Inc., Palo Alto, California) or Bruker Avance AVII 600 MHz NMR spectrometer (Bruker BioSpin GmbH, Rheinstetten, Germany) equipped with a cryoprobe and using standard pulse sequences. All samples were dissolved in deuterated DMSO-*d6* or CDCl_3_.

### Antifungal susceptibility test

The antifungal activity of purpurfusarin was tested towards *Candida albicans* in accordance with the CLSI standards in RPMI-1640 medium adjusted to pH 7 with 0.165 M MOPS buffer^48^. The inoculated media (2.5 × 10^3^ cells per ml) was transferred to 96 well microtiter plates in aliquots of 200 μL using a Hamilton STAR liquid handling workstation with an integrated Thermo Cytomat shaking incubator and Biotek Synergy Mx microplate reader. The test compounds were dissolved in DMSO and applied in concentrations ranging from 100 μM to 1.25 μM (Holm *et al*.^29^). The plates were incubated at 35 °C in an integrated shaking incubator under constant shaking at 1200 rpm with an amplitude of 2 mm. Optical density was automatically recorded every hour for 20 hours using the integrated plate-reader.

## Additional Information

**How to cite this article**: Frandsen, R. J. N. *et al*. Black perithecial pigmentation in *Fusarium* species is due to the accumulation of 5-deoxybostrycoidin-based melanin. *Sci. Rep.* **6**, 26206; doi: 10.1038/srep26206 (2016).

## Supplementary Material

Supplementary Information

## Figures and Tables

**Figure 1:**
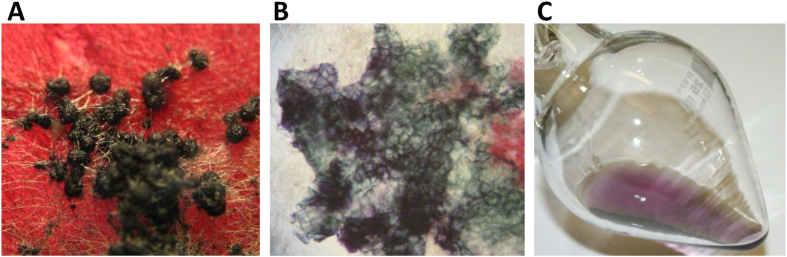
*Fg* perithecia. **(A)** *Fg* wild type on carrot agar. **(B)** Crushed perithecium showing the purple pigmentation of the periderm - the vegetative mycelium (red) is visible to the right. **(C)** Purified purpurfusarin in DMSO.
