# Supplementary material for: Black perithecial pigmentation in Fusarium species is due to the accumulation of 5-deoxybostrycoidin-based melanin
Source: Sci Rep. 2016 May 19;6:26206. doi: 10.1038/srep26206 (PMC4872168; doi:10.1038/srep26206)
Supplement: Supplementary Information [file srep26206-s1.pdf]

SUPPLEMENTARY INFORMATION for the article

"Black perithecial pigmentation in *Fusarium* species is due to the accumulation of 5-deoxybostrycoidin-based melanin"

Rasmus J.N. Frandsen*, Silas A. Rasmussen, Peter B. Knudsen, Silvio Uhlig, Dirk Petersen, Erik Lysøe, Charlotte H. Gotfredsen, Henriette Giese & Thomas O. Larsen

Contents

Prediction of the catalytic potential of the PGL1 gene cluster 2
Primers used for genetic modifications and validation 3
Genetic modification strategy 5
Validation of genetically modified strains by PCR and Southern blot analysis 7
Evolutionary models for the development of pigment biosynthesis in *Fusarium* 15
UV/VIS and MS data for compound (1) to (5) 16
NMR data for compound (3) and (5) 17
Possible configuration of purpurfusarin (5) 19
Selected HMBC and NOE correlation for purpurfusarin (5) 19
Purification scheme for compound (3), (4) and (5) 21

Prediction of the catalytic potential of the PGL1 gene cluster

Table S1 Prediction of the catalytic potential of the PGL1 gene cluster. Predictions based on the identified Pfam & CDD motifs, combined with the most similar characterized enzymes.

Name	Accession no	Identified structural domains	Predicted catalytic potential or function
PGL1	FGSG_17168	SAT (aa 1 - aa 369)<br>KS (cd00833)<br>AT (pfam00698)<br>PT (aa 1227 - aa 1576)<br>ACP (pfam00550)<br>ACP (pfam00550)<br>Dehydrogenase (COG3320)	Polyketide synthase
PglJ	FGSG_15669	O-methyltransferase (pfam00891)	O-methyltransferase (EC2.1.1.110)
PglM	FGSG_09184	FAD-binding monooxygenase (pfam01494)	Monooxygenase (EC 1.14.13.8)
PglL	FGSG_09185		Adenylosuccinate lyase (EC 4.3.2.2)
PglX	FGSG_17170	Zinc-binding dehydrogenase (pfam00107)	Dehydrogenase/quinone reductase (EC 1.6.5.5)
PglV	FGSG_09187	Short chain dehydrogenase (COG1028)	Short-chain dehydrogenase (EC 1.1.1.100)
PglR	FGSG_17171	Zn(II)$_2$Cys$_2$ transcription factor (cl00068)	Fungal transcription factor
PglE	FGSG_09189	Alpha/beta hydrolase family (pfam12697)<br>Esterase (cd12809)	Esterase

Primers used for genetic modifications and validation

Table S2 Primers used in the study. In application: S/V = Screening and validation

Gene	Sequence	gDNA	Application
PGL1-A1	5′- **TCGGTACCAAGGCCC**TAGGAGGTCAGCCTTTGTGATGCCACC	1895 bp	Replacement of *PGL1*
PGL1-A2	5′- **GGCCGTGGCCAGCCC**TGGACACCTGTCAACGACATCGCTGAT		
PGL1-A3	5′- **CGTTTAAACGGATTT**TCATTGCAAAACTCTTCAACGCCCTCA	1839 bp	Replacement of *PGL1*
PGL1-A4	5′- **GACATTAATTAATTT**GCGCCAAGCAAATGCTCGACATTAGAG		
PGL1-T3	5′- TCGCTTTCGCAGCGGGCTTGATAA	2327 bp	S/V
PGL1-T4	5′- CGGCCCTTGATCTCCTCTGGTAGGTCT	2237 p	S/V
PGL1-O1	5′- **GACATTAATTAATTT**CGAGGTGGGATAAAAATACTTACATC	1294 bp	Overexpression of *PGL1*
PGL1-O2	5′- **CGTTTAAACGGATTT**AACAAAGGTGTTGAATCTTGTAAAG		
PGL1-O3	5′- **GAGCAGACATCACCC**ATGGCTTCCCATATTAAGCTGTATCTC	1222 bp	Overexpression of *PGL1*
PGL1-O4	5′- **TCGGTACCAAGGCCC**AACGATAGCAAGCTTTGGCTTGGCGG		
PGL1-OT1	5′- ACTGAACGCATGCCACGACAACTCATC	2044 bp	S/V
PGL1-OT2	5′- CGAAGACTTGGGACTGGCTTGTGAACA	1569 bp	S/V
PGLJ-U1	5´- GGGTTTAAUAGCTTGGCTGAGCTTGTGTTAGTGA	1001 bp	Replacement of *PGLJ*
PGLJ-U2	5′- GGACTTAAUCATCTTGGGTTAGGTTATGTTGTATGCA		
PGLJ-U3	5′- GGCATTAAUTAGATTGGGGTAGATTGGGGTAGATTTG	1003 bp	Replacement of *PGLJ*
PGLJ-U4	5′- GGTCTTAAUACTGGCAGCTCAAGTTCAGAGATCC		
PGLJ-T1	5′- CCATCTCTTACGATCAAGCCGC	579 bp	S/V
PGLJ-T2	5′- GGGAGGTCTTCAATCACGAAGTTG		
PGLJ-T3	5′- TGCCAGCCAAGTATTGTGCAAG	1573 bp	S/V
PGLJ-T4	5′- AGAACAACGCAGTGCACCCAGC	1208 bp	S/V
PGLM-U1	5´- GGGTTTAAUTCATGTTAGCCACAGCATTTCTCAATC	1000 bp	Replacement of *PGLM*
PGLM-U2	5′- GGACTTAAUTAAGCGAGTTTAGGGGAGTGTTAGGG		
PGLM-U3	5′- GGCATTAAUCATATCTTGGGCATGCGTTTTCTC	1009 bp	Replacement of *PGLM*
PGLM-U4	5′- GGTCTTAAUGCGGTTTATTATGTGCAGAATCTCAAA		
PGLM-T1	5´- TTGCGAGAGTGACGCCGTCTTCAAT	520 bp	S/V
PGLM-T2	5´- GCGTTGAAGGACCCTTTGGTCA		
PGLM-T3	5´- TATCATGGCTGCGCTGAAAACC	1474 bp	S/V
PGLM-T4	5´- GCTGCTGAGTTGAATGTTTAAAGAGC	1281 bp	S/V
PGLX-U1	5´- GGGTTTAAUAATCTCAAGGAAACTGGCCT	1000 bp	Replacement of *PGLX*
PGLX-U2	5′- GGACTTAAUTGATTGAAATACTTTTCAATAATAT		
PGLX-U3	5′- GGCATTAAUAGAGCTGTTCTGTTCGACAGACGATAA	1039 bp	Replacement of *PGLX*
PGLX-U4	5′- GGTCTTAAUTGAGGCTTCTTCATTCACCGTCG		
PGLX-T1	5´- GCTGAAGTATCTGGTAAAAACATATCCAAAG	553 bp	S/V
PGLX-T2	5´- AACATGGCTGACGATGATCACA		
PGLX-T3	5´- GTAACACTGGTACCAAAACCAGTAACAGCGTTG	1300 bp	S/V
PGLX-T4	5´- TAGCTCCTGTTCGCGTCAACGC	1056 bp	S/V
PGLV-U1	5´- GGGTTTAAUTCATACACTCCTTCGCCAACCTCA	1000 bp	Replacement of *PGLV*
PGLV-U2	5′- GGACTTAAUTAGAACGGAGAGACTGGTCTCCGA		
PGLV-U3	5′- GGCATTAAUCATTTTGATGATTTGAAGTTTCAATTG	1028 bp	Replacement of *PGLV*
PGLV-U4	5′- GGTCTTAAUCGCAATTATCTTGACATACAATATTGGC		
PGLV-T1	5´- ACATCCTCCACCTGGCCAATCG	500 bp	S/V
PGLV-T2	5´- ATCCCTTGCAACCTCGGCTCCT		
PGLV-T3	5´- CAGAGTCTGGGACTGGAAGCCA	1416 bp	S/V
PGLV-T4	5´- GCGGTGAGGAATATGACGGAAG	1354 bp	S/V
PGLR-E1	5′- **GGACTTAAU**GTCTGACGGCTATCGAATTTTCGA	2196 bp	Overexpresion of *PGLR*
PGLR-E2	5′- **GGGTTTAAU**GAGTGTTGATGCCATTTTGATGATTTG		
RF-1	5′- AAATTTTGTGCTCACCGCCTGGAC		S/V
RF-2	5′- TCTCCTTGCATGCACCATTCCTTG		S/V
RF-3	5′- TTGCGTCAGTCCAACATTTGTTGCCA		S/V
Hyg588 U	5′- AGCTGCGCCGATGGTTTCTACAA	588 bp	Screening
Hyg588 L	5′- GCGCGTCTGCTGCTCCATACAA		

Table S3 Primers used for RT-PCR

Gene	Sequence	gDNA	cDNA	Application
FG09177 upper	5′- CGGAAAACTCATCCCAGAACTCGTGGC	534 bp	534 bp	RT-PCR
FG09177 lower	5′- TGCCTATCGTTTCACATGCTTCCCAGA			
FG09178 upper	5′- ATAAGTAACGACGGCAACGGAGACGGC	545 bp	545 bp	RT-PCR
FG09178 lower	5′- TCATCAATCAAAAGCTCGCGGAATCG			
FG09179 upper	5′- AGAAGGTCCTAACCCCCGTGTTGGCGT	502 bp	367 bp	RT-PCR
FG09179 lower	5′- CGTCTGCCCCAGCAAATTCACCAA			
FG09180 upper	5′- TTCTTCAGCTTGGAAAACTGTGCCCCG	473 bp	473 bp	RT-PCR
FG09180 lower	5′- CGTTTCTTTTGCGCCAAGTTGTTCGA			
FG09181 upper	5′- ATCTGCGTTGTACCCTGACATTCCGCG	496 bp	496 bp	RT-PCR
FG09181 lower	5′- CGTATCCTCTTCCACGTATGCCACCGA			
Fgd371-210 upper	5′- ACGGGACTAACAGTCGAGCATCAGGGC	496 bp	496 bp	RT-PCR
Fgd371-210 lower	5′- TCCTGGTCAACAAGCGCATCTCCGT			
PGL1-T1 (CDS)	5′- GTTTGACCAGCTGCGAGACCCCATTG	541 bp	541 bp	RT-PCR and validation
PGL1-T2 (CDS)	5′- TCGTCCACAAGTATGCCGCTGGCTT			
FG09183 upper	5′- TTCGTGATTGAAGACCTCCCAGAGCCC	493 bp	493 bp	RT-PCR
FG09183 lower	5′- TGAACGCATGCCACGACAACTCATCTA			
FG09184 upper	5′- GCCCAATCTCCATCTTGCAAATCCTCC	528 bp	528 bp	RT-PCR
FG09184 lower	5′- GCAGCTCAAGTTCAGAGATCCCCACGA			
FG09785 upper	5′- TTCAACTCAGCAGCTTTGGCCTTCTGG	602 bp	479 bp	RT-PCR
FG09785 lower	5′- TGGAGCGATCTCTGGATGATTCCGC			
FG09186 upper	5′- CCCCTGCTGAATACCTTCCAATCCACC	593 bp	491 bp	RT-PCR
FG09186 lower	5′- CACTCACAGCACTAACTGCGGCATGTG			
FG09187 upper	5′- CATAAGCCTCAGCCACATCCTCCACCT	491 bp	491 bp	RT-PCR
FG09187 lower	5′- CGAGACCCTCAACTCGGAAGTCGAAAA			
PGLR-T1 (CDS)	5′- TGATCGGTCTCAACACAACGCCTTCTG	561 bp	561 bp	RT-PCR
PGLR-T2 (CDS)	5′- TGGCTTGGGATTACAGAGCATCGGAAT			
FG09189 upper	5´- CTGGCGTAAGAGGGGATCCGAT	472 bp	472 bp	RT-PCR
FG09189 lower	5´- GCCGCCCCATTCACAATAAGAA			
FG09190 upper	5´- TCCTGACATTACCTCCGACTACTATACC	458 bp	458 bp	RT-PCR
FG09190 lower	5´- CCTCCATAGCTTGCTCTACCGA			
FG09191 upper	5´- GACTCCATCTGACTCTCTTCATCTGAT	517 bp	517 bp	RT-PCR
FG09191 lower	5´- GGATGGAGAAGCATACAGAGAAGAAAT			
FG09192 upper	5′- TTATCCTTGCTTTAATGAGCTGGATTTTCC	608 bp	479 bp	RT-PCR
FG09192 lower	5′- CCTTCAAAGCTTCACCAGAAAAGACT			
FG09193 upper	5´- AAGACTGATGATGCTAGTTCCGTTTC	500 bp	500 bp	RT-PCR
FG09193 lower	5´- AGTACCCCGCATTCAGTAGGAAG			
FG09194 upper	5´- TTAGTCTAGCACCATAATACCAAGCCC	425 bp	359 bp	RT-PCR
FG09194 lower	5´- GCCAAAGTTATGCTTCTGGGGA			

Genetic modification strategy

Figure S1 Strategies for targeted replacement and overexpression of Gz-PGL1. The vectors were assembled in a single-step by In-Fusion PCR cloning. The validated plasmids were introduced in *Agrobacterium tumefaciens*, which mediated transformation of the T-DNA regions into *F. graminearum*. Targeted replacement of PGL1 (left in figure) was achieved by homologous recombination between the introduced T-DNA and the *PGL1 locus*, resulting in replacement of the *PGL1* coding sequence with the hygromycin resistance gene (*hph*). Targeted over expression (right in figure) was achieved by homologous recombination between the introduced T-DNA and the *PGL1* locus, resulting in integration of the *gapdh* promoter in front of the *PGL1* coding sequence to obtain constitutive expression of the *PGL1* in vegetative tissue and production of the primary product of the PKS.

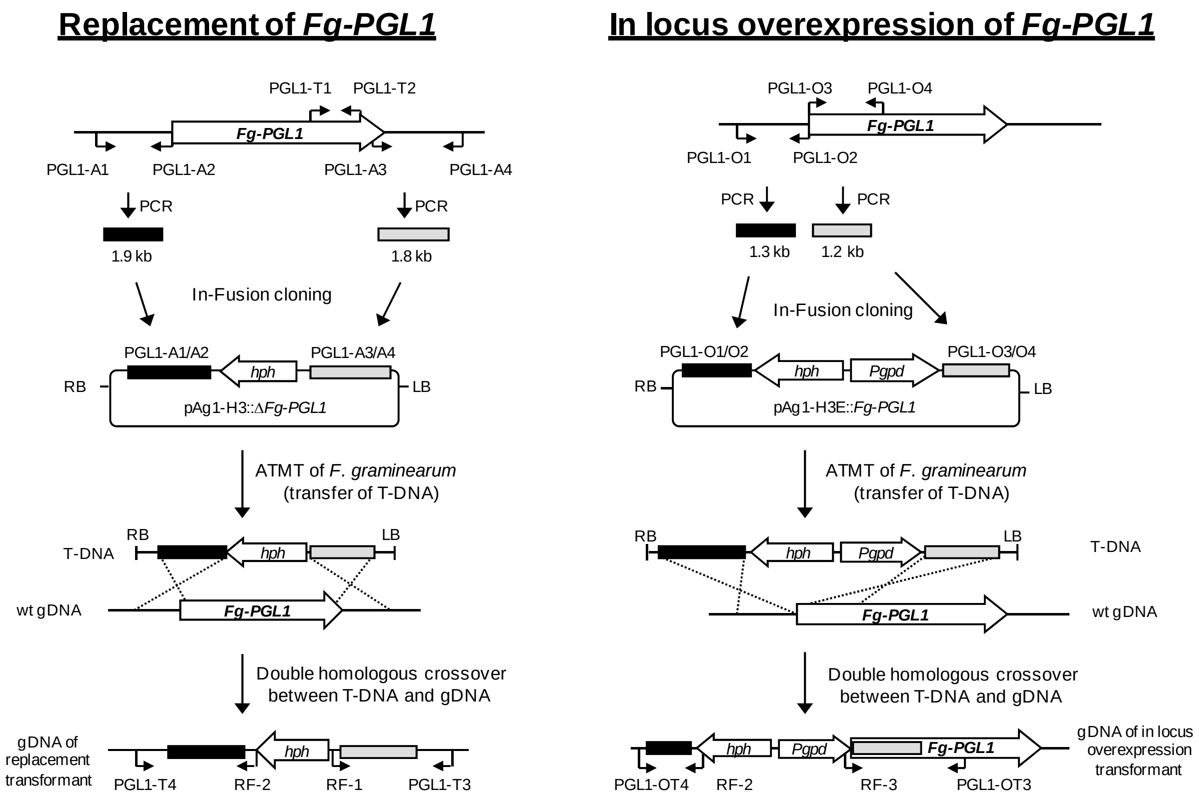

Figure S2 Strategy for ectopic overexpression of pglR. The PGLR coding sequence plus terminator was amplified by PCR, treated with USER enzyme mix and cloned into the expression vector pRF-HUE. The construct was introduced into the *F. graminearum* wild-type strain by *Agrobacterium tumefaciens* mediated transformation, where it was expected integrated randomly via the non-homologous end-joining pathway. However, only a single transformant was found to contain the full-length expression cassette integrated into the endogenous *pglR* locus. Targeted integration of the overexpression cassette into the *PKS12* locus resulted in the recovery of several transformants carrying the full-length construct.

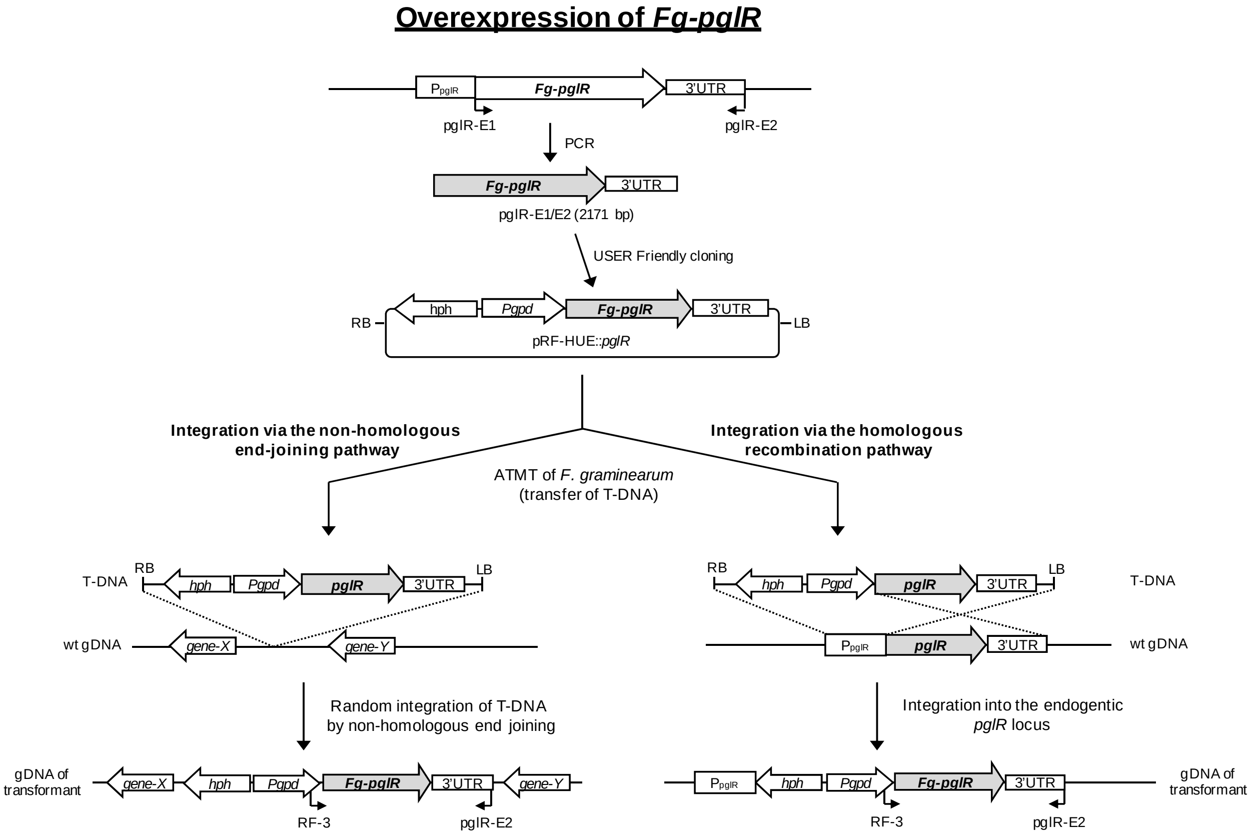

Validation of genetically modified strains by PCR and Southern blot analysis

Validation of the introduced genetic modifications was performed via diagnostic PCR followed by a Southern analysis.

The screening and validation process:

PCR validation:

The PCR screening and validation process consists of four different primer pairs for the gene deletion strains, three different primer pairs for the PGL1 *in locus* overexpression strain, and two primer pairs for the ectopic overexpression of *PGLR (PGL6)*. The aim of the PCR-based validation was to show that homologous recombination had occurred between both ends of the T-DNA and target locus in deletion and *in locus* overexpression strains. The used primers are described in Table S3 while the expected amplicon sizes for the GMO strain and the wild type are shown below.

Southern blot strategy:

Genetically modified strains that showed the expected results in the PCR screening were subjected to a Southern blot analysis to determine the T-DNA copy number. The "Vector NTI" software package was used to design Southern blot strategy for the individual GMO strains. A 588 bp long PCR amplified fragment of the hygromycin resistance gene was used as probe.
The two included figures shows the location of genes (above the line), primers (below the line) and restriction sites (above the line).

Southern blot analysis:

The aim of the Southern blot analysis was primarily to determine the T-DNA copy number and secondly to provide evidence for correct integration of the T-DNA. A probe targeting only the selection marker gene (hygR) was used. This strategy only results in bands in the genetically modified strains and not the wild type, as the wild type does not contain the selection marker gene.
The sizes of the obtained bands have been calculated based on their individual traveling length compared to that of the bands found in the utilized 2Log ladder, which is not shown on the blots.

ΔPGL1 strain

PCR validation

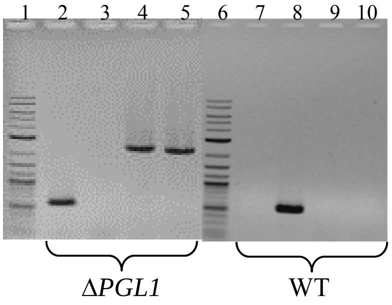

Lane	Sample	Result	Strain
1	2 log ladder		
2	Hyg588U/L	588 bp	ΔPGL1
3	PGL1-T1/T2	No band	ΔPGL1
4	PGL1-T3/RF-1	2327 bp	ΔPGL1
5	PGL1-T4/RF-2	2237 bp	ΔPGL1
6	2 log ladder		
7	Hyg588U/L	No band	WT
8	PGL1-T1/T2	541 bp	WT
9	PGL1-T3/RF-1	No band	WT
10	PGL1-T4/RF-2	No band	WT

Southern blot strategy:

Theoretical digestion of gDNA from the wild type and mutant strain with *SnaBI*

Wild type:

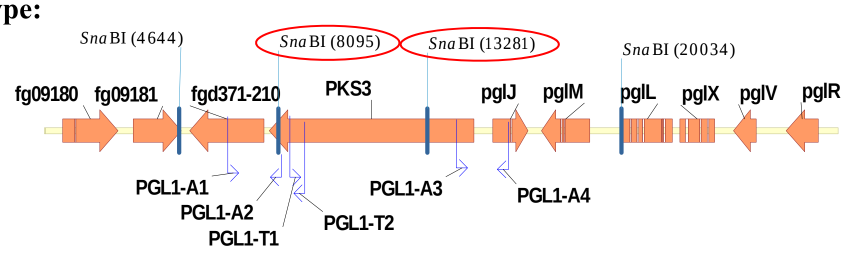

Mutant:

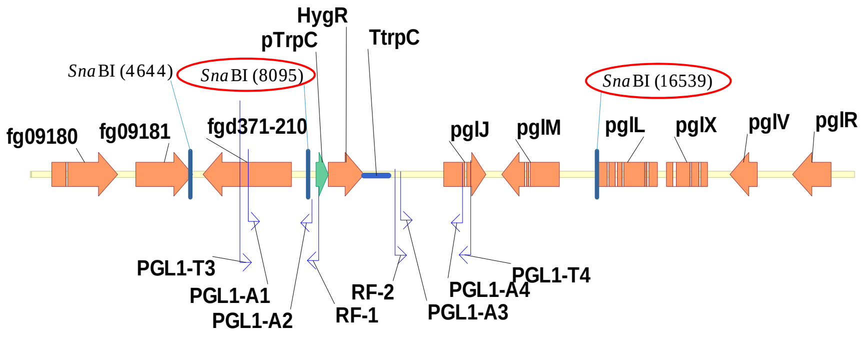

Expected band pattern

Enzym	WT	*ΔPGL1*
SnaBI	5186 bp	11895 bp

Southern blot analysis with *SnaBI* digested gDNA

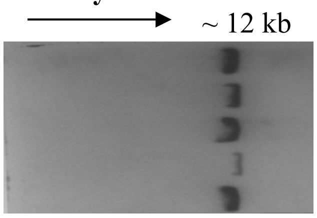

ΔPGL1 T1 *Correct*
ΔPGL1 T2 *Correct*
ΔPGL1 T14 *Correct*
ΔPGL1 T21 *Correct*
ΔPGL1 T32 *Correct*

Conclusion for the *ΔPGL1* strain

The isolated transformants displayed the expected band pattern in the PCR analysis. Southern blot analysis confirmed that the T-DNA had integrated by double homologous recombination, based on the hybridization band of ~12 kb. The Southern blot in addition showed that only a single copy had integrated.

PGL1 strain

PCR validation

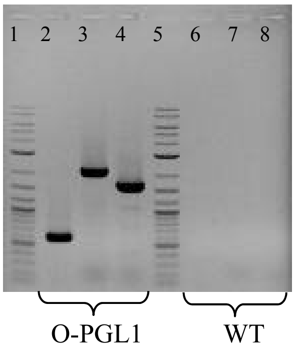

1	2 log ladder		
2	Hyg588U/L	588 bp	O-PGL1
3	PGL1-OT3/RF-3	2044 bp	
4	PGL1-OT4/RF-2	1569 bp	
5	2 log ladder		
6	Hyg588U/L	No band	WT
7	PGL1-OT3/RF-3	No band	
8	PGL1-OT4/RF-2	No band	

Southern blot strategy:

Theoretical digestion of gDNA from the wild type and mutant strain with *SnaBI*

Wild type:

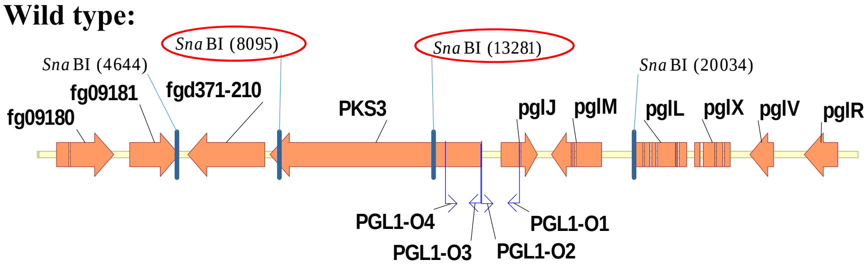

Mutant:

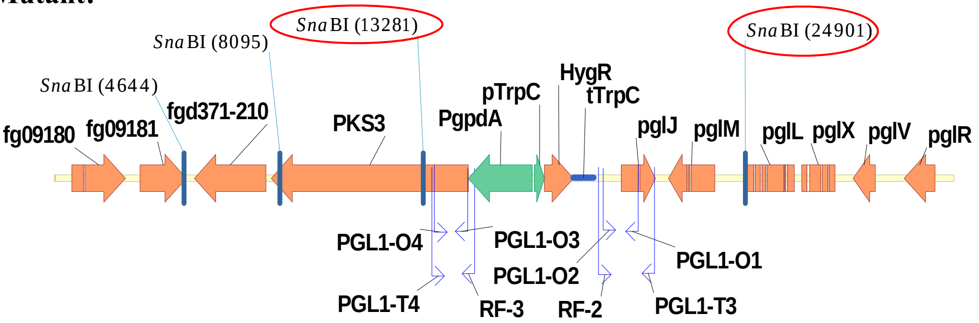

Expected band pattern

Enzym	WT	*ΔPGL1*
SnaBI	6753 bp	11620 bp

Southern blot analysis with *SnaBI* digested gDNA

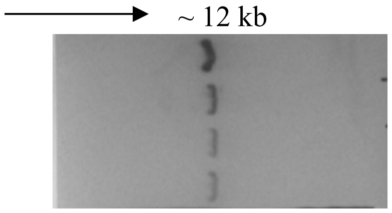

O-*PGL1* #OT1	*Correct*
O-*PGL1* #OT2	*Correct*
O-*PGL1* #OT17	*Correct*
O-*PGL1* #OT43	*Correct*

Conclusion for the *O-PGL1* strain

The isolated transformants displayed the expected band pattern in the PCR analysis. Southern blot analysis confirmed that the T-DNA had integrated by double homologous recombination, based on the hybridization band of ~12 kb. The Southern blot in addition showed that only a single copy had integrated.

ΔPGLJ strain

PCR validation

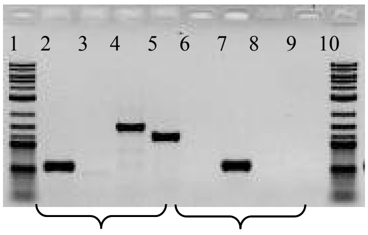

Lane	Primers	Result	
1	2 log ladder		
2	Hyg588U/L	588 bp	*ΔPGLJ*
3	PGLJ-T1/T2	No band	*ΔPGLJ*
4	PGLJ-T3/RF-1	1474 bp	*ΔPGLJ*
5	RF-2/PGLJ-T4	1281 bp	*ΔPGLJ*
6	Hyg588U/L	No band	WT
7	PGLJ-T1/T2	520 bp	WT
8	PGLJ-T3/RF-1	No band	WT
9	RF-2/PGLJ-T4	No band	WT
10	2 log ladder		

Southern blot strategy:

Theoretical digestion of gDNA from the wild type and mutant strain with *SnaBI*

Wild type:

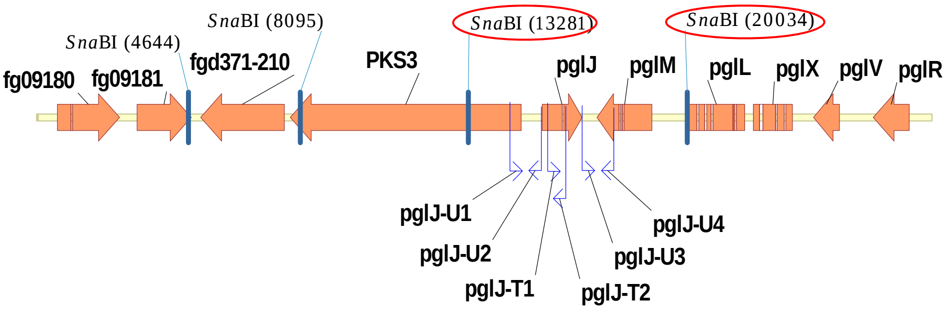

Mutant:

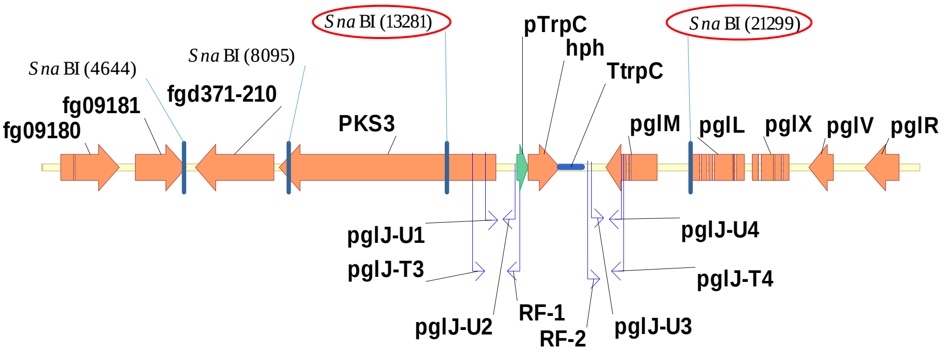

Expected band pattern

Enzym	WT	*ΔPGLJ*
SnaBI	6753 bp	8048 bp

Southern blot analysis with *SnaBI* digested gDNA

→ ~ 8 kb

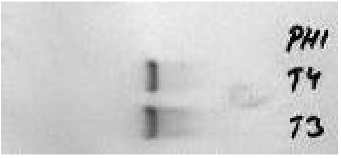

G. zeae PH-1 wild-type	
ΔPGLJ T4	*correct*
ΔPGLJ T3	*correct*

Conclusion for the *ΔPGLJ* strain

The isolated transformants displayed the expected band pattern in the PCR analysis. Southern blot analysis confirmed that the T-DNA had integrated by double homologous recombination, based on the hybridization band of 8 kb. The Southern blot in addition showed that only a single copy had integrated.

ΔPGLM strain

PCR validation

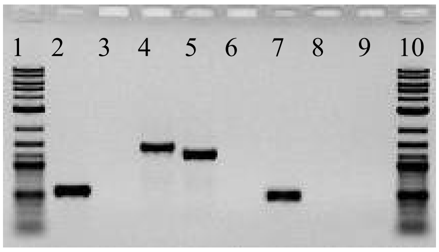

Δ*plg3* WT

1	2 log ladder		
2	Hyg588U/L	588 bp	Δ*PGLM*
3	PGLM-T1/T2	No band	
4	PGLM-T3/RF-1	1474 bp	
5	RF-2/PGLM-T4	1281 bp	
6	Hyg588U/L	No band	WT
7	PGLM-T1/T2	520 bp	
8	PGLM-T3/RF-1	No band	
9	RF-2/PGLM-T4	No band	
10	2 log ladder		

Southern blot strategy:

Theoretical digestion of gDNA from the wild type and mutant strain with *SnaBI*

Wild type:

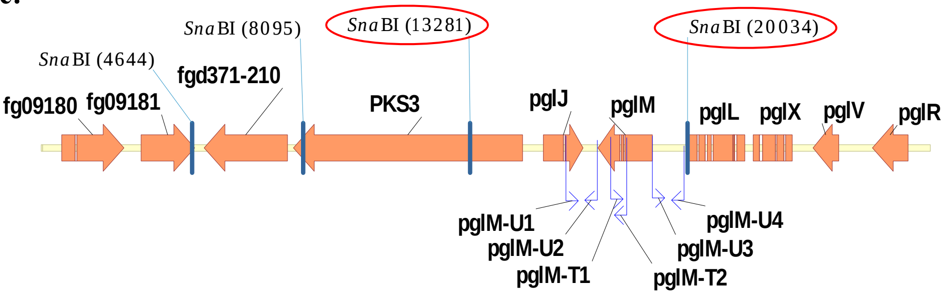

-

Mutant:

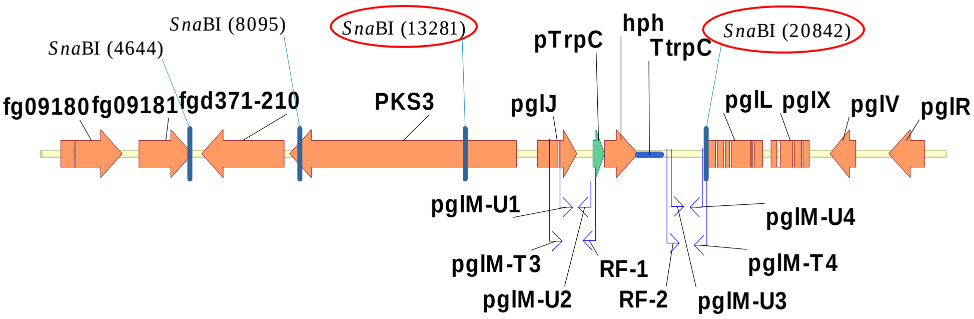

Expected band pattern

Enzym	WT	*ΔPGLM*
SnaBI	6753 bp	7561 bp

Southern blot analysis with *SnaBI* digested gDNA

→ ~ 7.5 kb

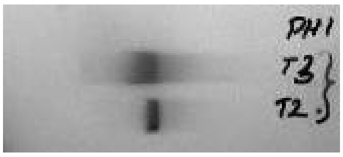

Gz PH-1 wild-type		
ΔPGLM T3	*double insert*	
ΔPGLM T2	*correct*	

Conclusion for the *ΔPGLM* strain

The isolated transformants displayed the expected band pattern in the PCR analysis. Southern blot analysis confirmed that the T-DNA had integrated by double homologous recombination, based on the hybridization band of 7.5 kb. The Southern blot in addition showed that only a single copy had integrated.

ΔpglX strain

PCR validation

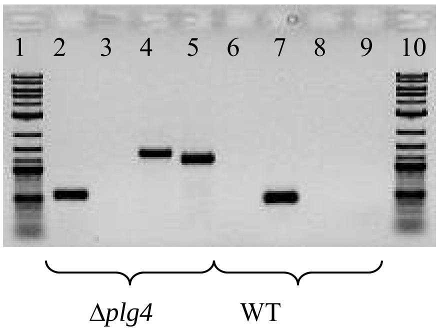

1 2 log ladder
2 Hyg588U/L 588 bp
3 pglX-T1/T2 No band
4 pglX-T3/RF-1 1300 bp
5 RF-2/PGLX-T4 1056 bp
ΔPGLX
6 Hyg588U/L No band
7 PglX-T1/T2 553 bp
8 pglX-T3/RF-1 No band
9 RF-2/PGLX-T4 No band
WT
10 2 log ladder

Southern blot strategy:

Theoretical digestion of gDNA from the wild type and mutant strain with *HindIII*

Wild type:

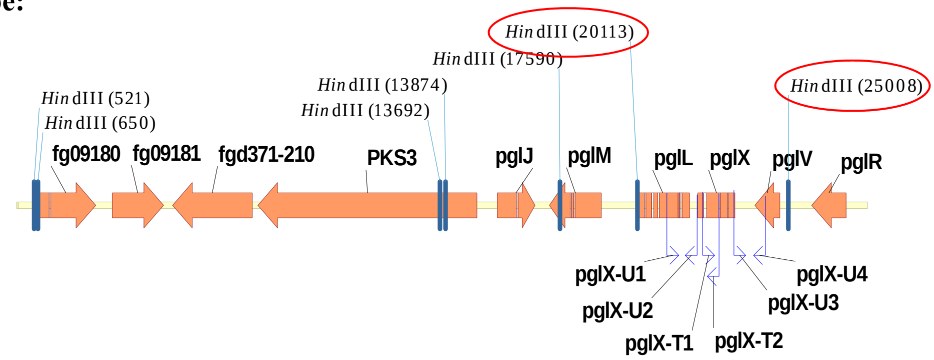

Mutant:

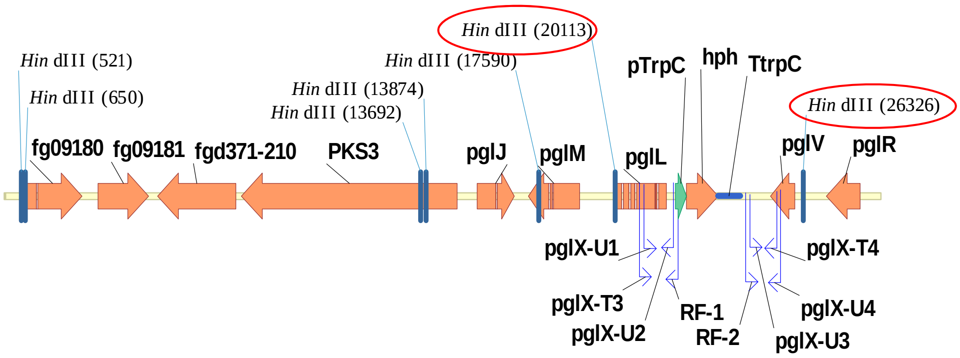

Expected band pattern

Enzym	WT	*ΔPGLX*
HindIII	4895 bp	6213 bp

Southern blot analysis with *HindIII* digested gDNA

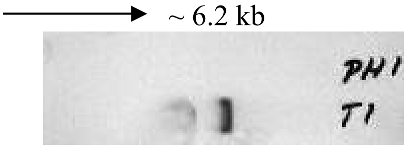

G. zeae PH-1 wild-type

ΔPGLX T1 *correct*

Conclusion for the *ΔpglX* strain

The isolated transformants displayed the expected band pattern in the PCR analysis. Southern blot analysis confirmed that the T-DNA had integrated by double homologous recombination, based on the hybridization band of 6.2 kb. The Southern blot in addition showed that only a single copy had integrated.

ΔPGLV strain

PCR validation

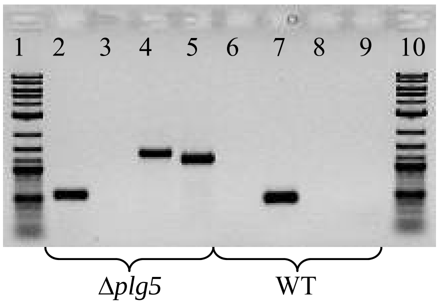

1	2 log ladder		
2	Hyg588U/L	588 bp	*ΔPGLV*
3	PGLV-T1/T2	No band	*ΔPGLV*
4	PGLV-T3/RF-1	1416 bp	*ΔPGLV*
5	RF-2/PGLV-T4	1354 bp	*ΔPGLV*
6	Hyg588U/L	No band	WT
7	PGLV-T1/T2	500 bp	WT
8	PGLV-T3/RF-1	No band	WT
9	RF-2/PGLV-T4	No band	WT
10	2 log ladder		

Southern blot strategy:

Theoretical digestion of gDNA from the wild type and mutant strain with *SalI*

Wild type:

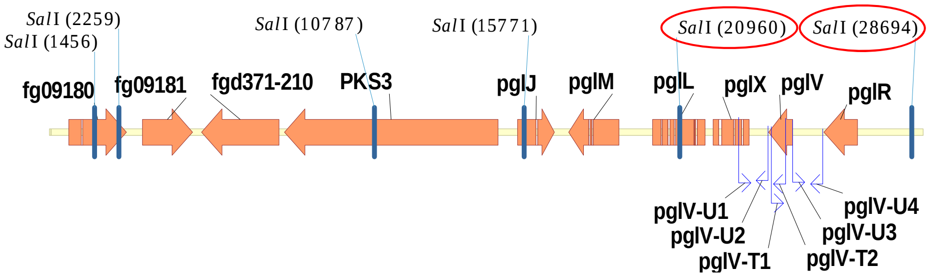

Mutant:

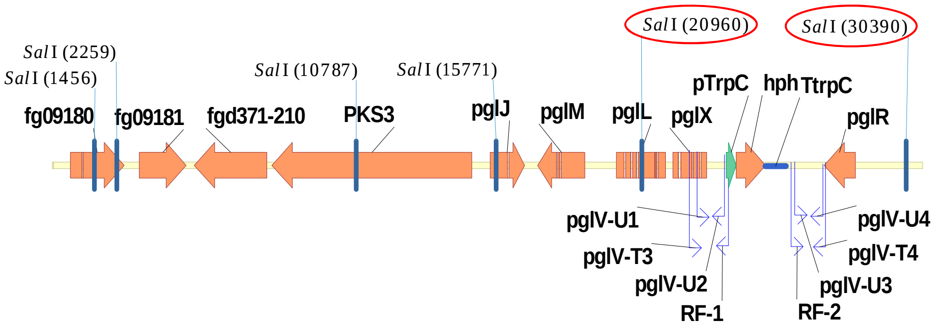

Expected band pattern

Enzym	WT	*ΔPGLV*
SalI	7734 bp	9530 bp

Southern blot analysis with *SalI* digested gDNA

→ ~ 9.5 kb

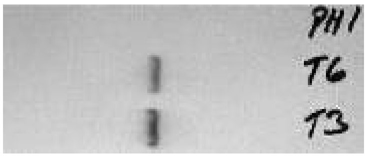

G. zeae PH-1 wild-type		
ΔPGLV T6		*correct*
ΔPGLV T3		*correct*

Conclusion for the *ΔPGLV* strain

The isolated transformants displayed the expected band pattern in the PCR analysis. Southern blot analysis confirmed that the T-DNA had integrated by double homologous recombination, based on the hybridization band of 9.5 kb. The Southern blot in addition showed that only a single copy had integrated.

PGLR strain

PCR validation

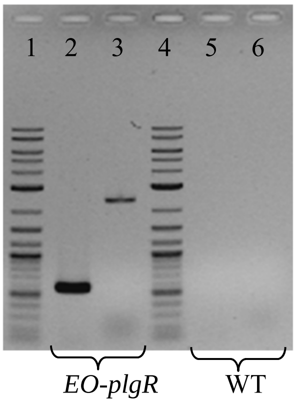

1	2 log ladder		
2	Hyg588U/L	588 bp	O-PGL1
3	RF-3/pglR-E2	2171 bp	O-PGL1
4	2 log ladder		
5	Hyg588U/L	No band	WT
6	pglR-OT3/RF-3	No band	WT

Southern blot strategy:

The ectopic integration of the T-DNA makes it impossible to predict the fragment sizes obtained during the Southern blot analysis. We therefore chose to cut the genomic DNA with BstxI, which does not cut internally in the T-DNA. Based on this one would expect two bands when probing with the coding sequence of PGLR fragment (one from the endogenetic copy and one from the introduced copy)

Southern blot analysis with *SnaBI* digested gDNA

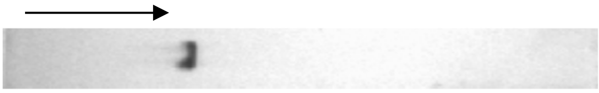

~ 7 kb

EO-PGLR (PGL6) T2 *Single insert*

Conclusion for the *EO-PGLR (PGL6)* strain

The isolated transformant displayed the expected band pattern in the PCR analysis. Southern blot analysis using part of the pglR CDS as a probe showed that the strain only contained a single copy of the gene. This suggests that the T-DNA copy has integrated by a single homologous recombination event with the endogenic locus.

Evolutionary models for the development of pigment biosynthesis in *Fusarium*

The Figure S3 below shows the possible models for the evolution of pigment biosynthesis in the *Fusarium* genus. Loss of a gene cluster is indicated by '– 'in red, while gains (horizontal gene transfers) are indicated by '+' in green. The expression pattern of the individual cluster is indicated by 'm' for mycelium and 'p' for perithecial. '*' represent shifts in the expression pattern of the *PGL1* gene cluster following introduction of a redundant gene cluster.

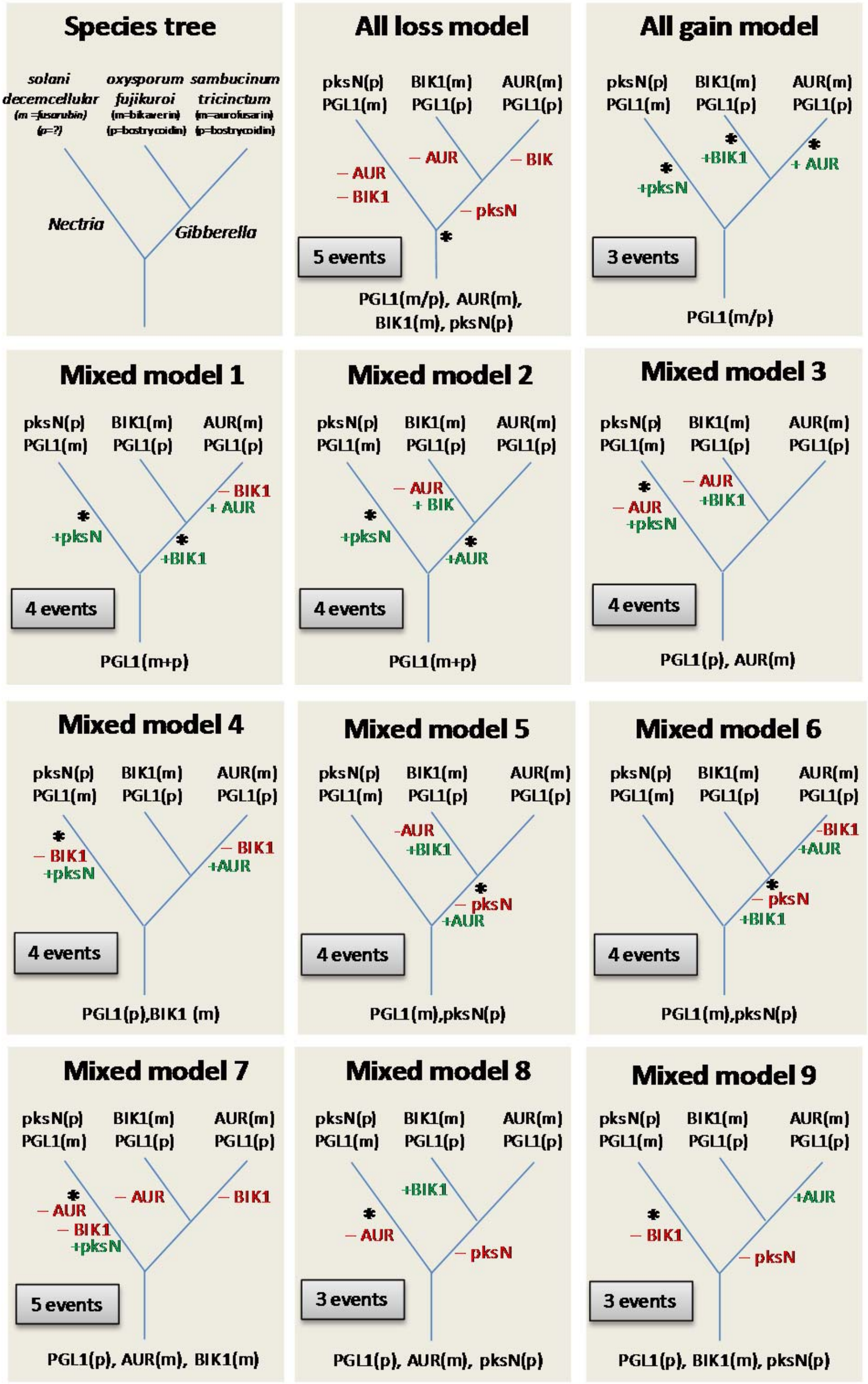

UV/VIS and MS data for compound (1) to (5)

	UV	*m/z* of $[M+H]^+$	Elemental composition
**(1)**	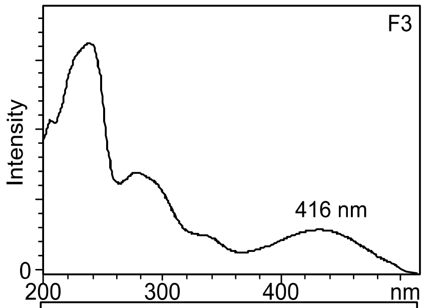	256.0604	$C_{14}H_9NO_4$
**(2)**	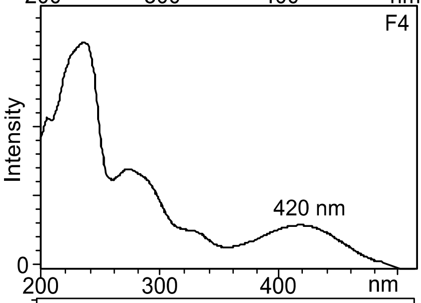	270.0761	$C_{15}H_{11}NO_4$
**(3)**	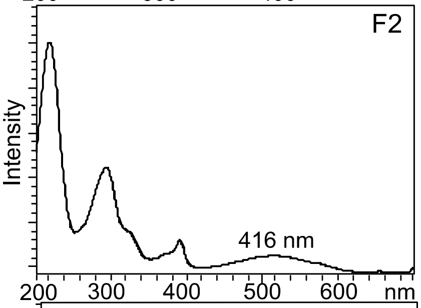	256.0965	$C_{15}H_{13}NO_3$
**(4)**	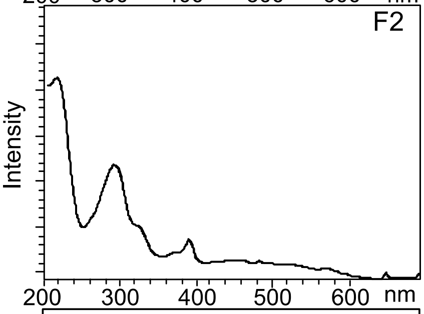	242.0812	$C_{14}H_{11}NO_3$
**(5)**	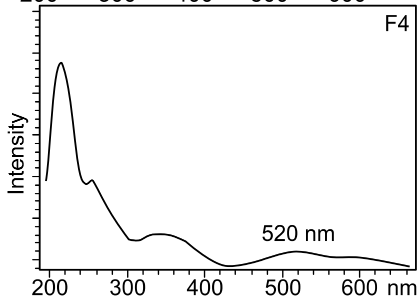	477.1083	$C_{28}H_{16}N_2O_6$

NMR data for compound (3) and (5)

Table S4 ^{1}H-^{13}C correlations (δ in DMSO-d_6) observed in the HMBC spectra of 5-deoxybostrycoidin anthrone (**3**).

#No	δ ^{1}H (Multiplicity)	δ^{13}C	HMBC
15	2.64 (s)	24.1	3, 4
16	3.87 (s)	54.9	6
10	4.24 (s)	31.5	1, 4, 5, 11, 12 , 14
7	6.44 (s, *J*=1.8)	98.9	5, 8
5	6.46 (s, *J*=1.8)	105.8	12
4	7.17 (s)	121.6	3, 10, 14, 15
1	9.35 (s)	148.5	3, 14
OH-8	13.17 (s)	-	12
12	-	110.9	
3	-	162.1	
14	-	124.2	
11	-	141.7	
13	-	148.5	
6	-	165.6	
8	-	165.9	
9	-	n.o.	

Table S5. ^{1}H and ^{13}C NMR and assignments and ^{1}H-^{13}C correlations (δ in DMSO-d_6) observed in the HMBC spectra for purpurfusarin (**5**). ^{*1}H and ^{13}C NMR shifts relative to $(CHD_2)_2SO$ = 2.49 ppm and $(CD_3)_2SO$ = 39.5 ppm, respectively. (w) refers to a weak correlation observed in a 3 Hz optimized HMBC experiment.

#No	δ ^{1}H (Multiplicity)	δ^{13}C	Integral	HMBC
15, 15'	2.37 (s)	23.5	6	3, 4
7, 7'	6.39 (s)	104.2	2	5, 6, 8, 12
4, 4'	7.72 (s)	123.6	2	13, 14, 15
1, 1'	9.43 (s)	147.3	2	3, 9, 14, 13,
OH-8, OH-8'	15.86 (s)	-	2	6, 7, 8, 9, 12
OH-6, OH-6'	18.43 (s)	-	1	6, 7
12, 12'	-	100.3		
5, 5'	-	120.6		
14, 14'	-	122.1		
10, 10'	-	125.5		
11,11'	-	130.0		
13, 13'	-	140.1		
3, 3'	-	157.8		
8, 8'	-	169.1		
6, 6'	-	173.4		
9, 9'	-	180.6		

Possible configuration of purpurfusarin (5)

The oxidative coupling of two anthrones could give rise to 2 different isomeric configurations, C1 and C2, as shown in the figure below. The NOESY spectrum shows a correlation between H-15 and H-1 and H-4 and it did not show any correlation between H-4 and H-7 that might have been expected if **5** had adopted the isomeric configuration C2. Hence, we expect (5) to adopt the C1 configuration.

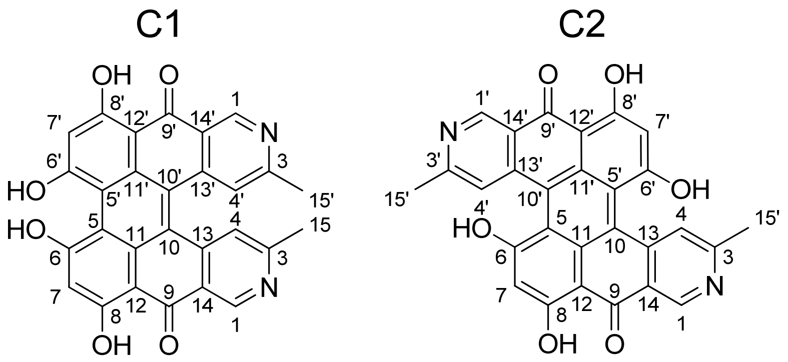

Selected HMBC and NOE correlation for purpurfusarin (5)

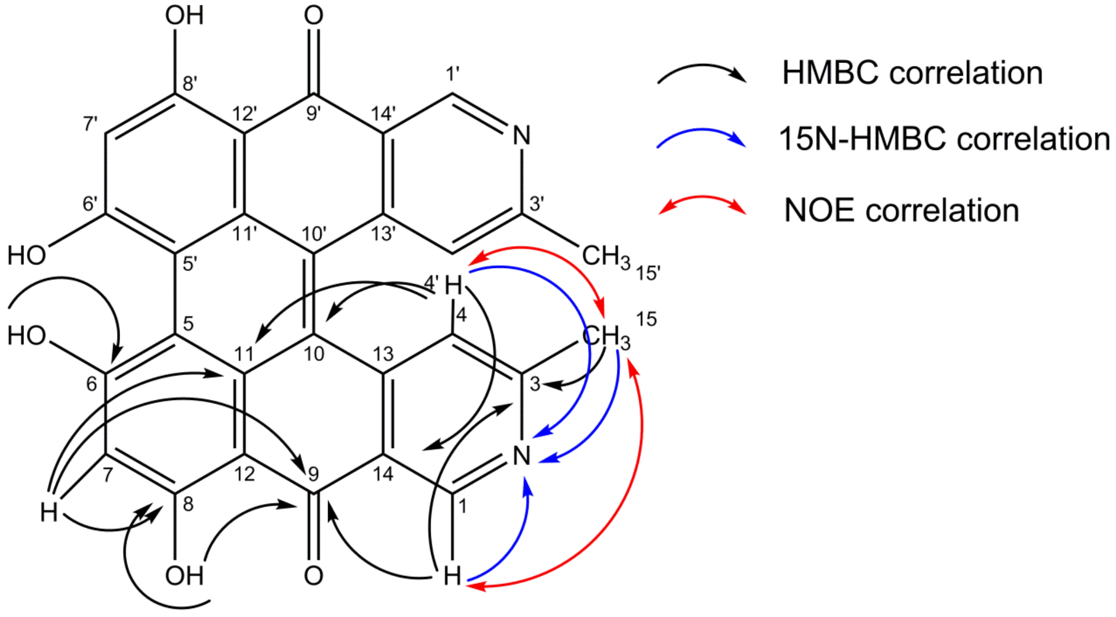

Pupurfusarin (**5**)

^{15}N-HMBC spectrum of pupurfusarin in MeOH-d4. The four protons H-1/H-1’ (δH = 9.43 ppm), H-4/H-4’ (δH = 7.72 ppm) and the protons of the methyl group H-15/H-15’ (δH = 2.73 ppm) all showed long-range correlation to a ^{15}N resonating at 317.5 ppm

15N-HMBC of (5)

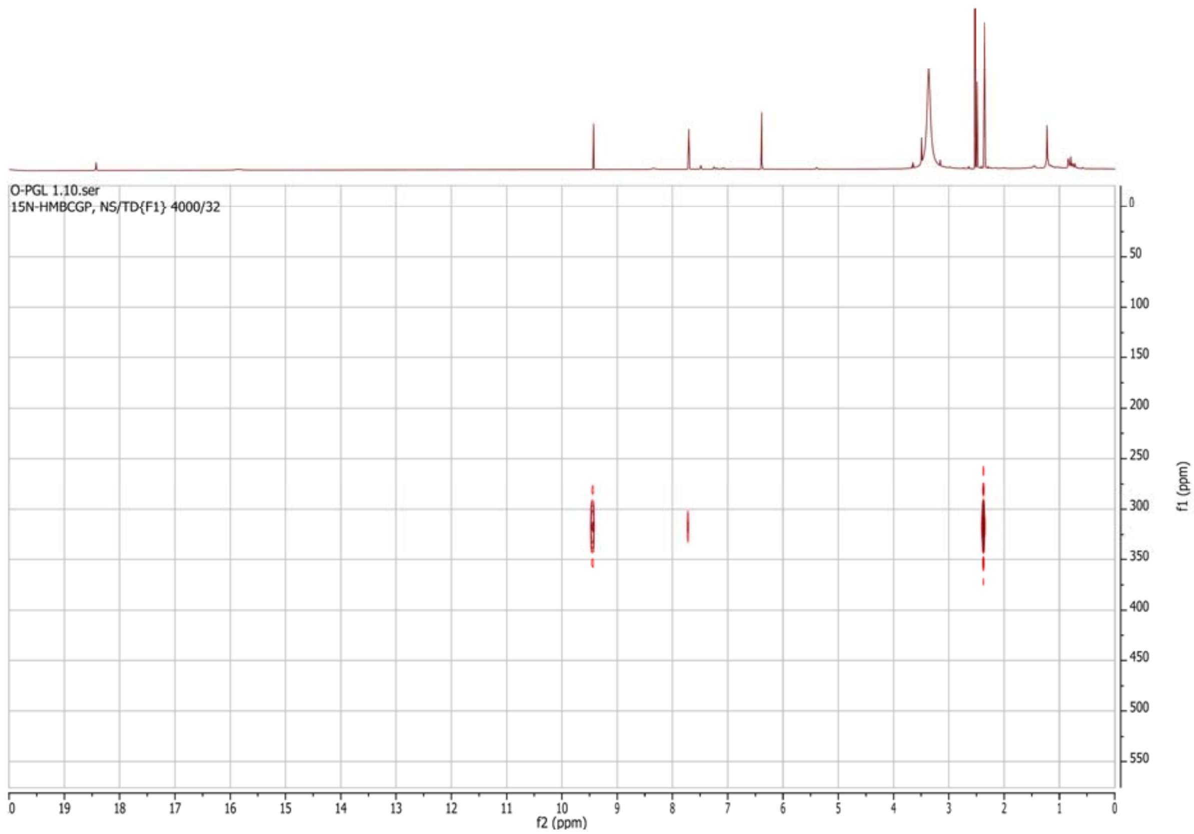

13CAPT of (5)

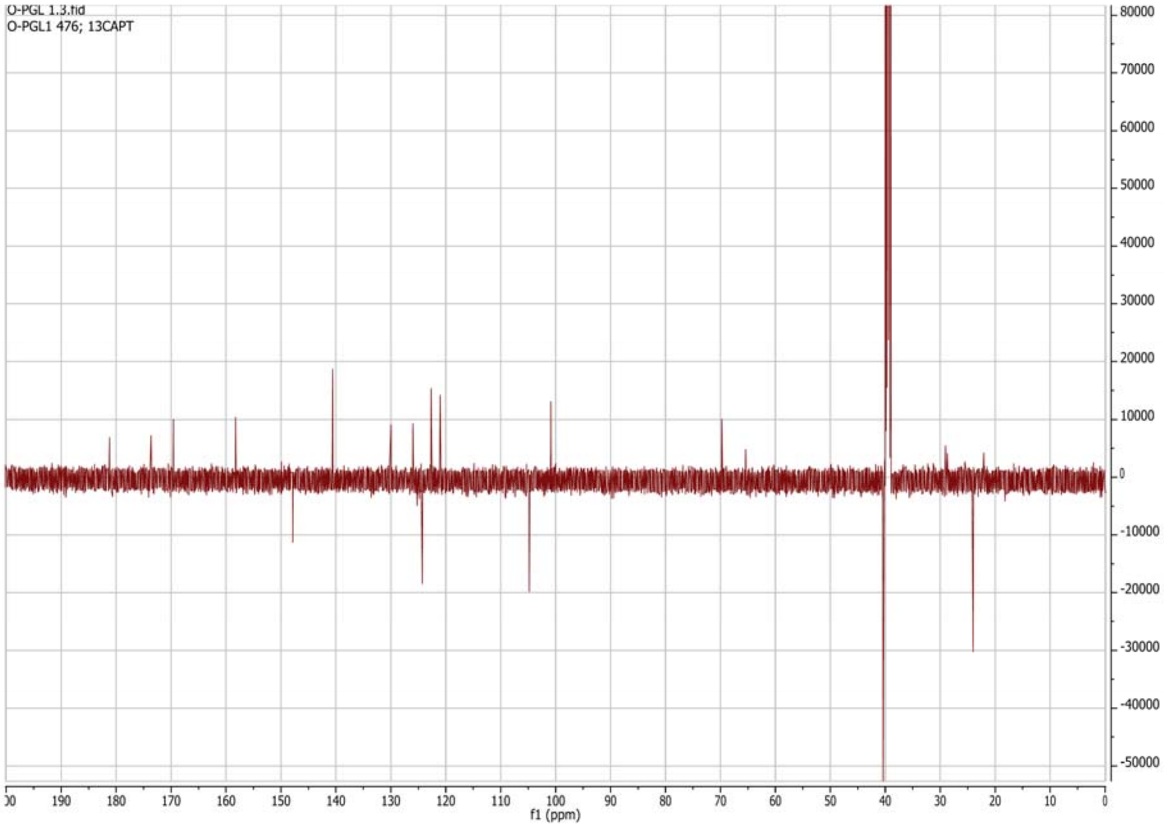

Purification scheme for compound (3), (4) and (5)

Table S6 purification of (**3**)

Compound	Fractionation	Semi-Prep
**(3)** 5-deoxybostrycoidin anthrone	No fractionation of crude extract	**(3)** was almost pure after the Liquid-Liquid extraction with DCM. The final purification of (3) was first attempted on LUNA C18 using a water-MeCN gradient with no success.<br>**(3)** was successfully purified using LUNA PFP using a water-MeOH gradient from 60-100 % containing 20 mM FA. The yield was 3.8 mg.

Table S7 purification of (**2**)

Compound	Fractionation	Semi-Prep
**(4)** 6-O-Demethyl-5-deoxybostrycoidin anthrone	10 g Isolute DIOL (Biotage, Uppsale, Sweden).<br>Eluted with the following solvent (30 ml):<br>1. DCM<br>2. DCM:EtOAc (1:1)<br>3. DCM:EtOAc (2:8)<br>4. EtOAc<br>**5. EtOAc:MeOH (95:5)**<br>**6. EtOAc:MeOH (8:2)**<br>7. EtOAc:MeOH (1:1)<br>8. EtOAc:MeOH (1:1)<br>9. MeOH<br>10. MeOH	The pooled fraction of the EtOAc:MeOH(95:5) and EtOAc:MeOH (8:2) 33.8 mg was further purified on LUNA PFP (250x10 mm, 5µm, Phenomenex, Torrance, CA, U.S.) using a 60-100 MeOH % + 20 mM FA gradient over 20 min. The yield of **(4)** was below 1 mg.

Table S8 purification of (**5**)

Compund	Fractionation	Semi-Prep
Pupurfusarin **(5)**	10 g Isolute DIOL (Biotage, Uppsale, Sweden).<br>Eluted with the following solvent (15 ml):<br>1. Heptane:DCM (1:1),<br>2. DCM:EtOAc (8:2),<br>3. DCM:EtOAc (4:6),<br>4. DCM:EtOAc (2:8),<br>5. EtOAc<br>6. EtOAc<br>7. EtOAc:ACN (8:2),<br>8. EtOAc:ACN (1:1),	LH-20 (40x4 cm) equilibrated in MeOH. The last five MeOH fractions from the second Diol fractionation was pooled and dried. The extract was dissolved in 400 ul MeOH and loaded on the column. The column was eluted with MeOH with a linear flow rate of 3.2 cm/hour. The Purple band was visually followed and eluted after 16 hours.

	9. EtOAc:ACN<br>10. EtOAc<br>11. EtOAc:ACN(8:2)<br>12. EtOAc:ACN (1:1)<br>13. EtOAc:ACN (1:1)<br>14. ACN<br>15. ACN<br>16. ACN<br>**17. 5x MeOH,**<br><br>The last five MeOH fractions was then dried and re-run on a 10 g Isolute DIOL (Biotage, Uppsala, Sweden) with the following solvents:<br>1. EtOAc<br>**2. EtOAc:ACN(8:2)**<br>**3. EtOAc:ACN (1:1)**<br>4. ACN<br>5. MeOH	
